# Normalization and cross-entropy connectivity in brain disease classification

**DOI:** 10.1016/j.isci.2025.112226

**Published:** 2025-03-17

**Authors:** Haifeng Wu, Shunliang Li, Yu Zeng

**Affiliations:** 1 School of Electrical and Information Technology, Yunnan Minzu University, Kunming 650504, China; 2 Yunnan Key Laboratory of Unmanned Autonomous System, Kunming 650504, China; 3 Multivariate Sensor Network & Information System of Science & Technology Innovation Team in University of Yunnan Province, Yunnan Minzu University, Kunming 650504, China

**Keywords:** Neuroscience, Mathematical biosciences, Biocomputational method

## Abstract

In resting-state functional magnetic resonance imaging (rs-fMRI), Pearson correlation has traditionally been the dominant method for constructing brain connectivity. This paper introduces an entropy-based connectivity approach utilizing subject-level *Z* score normalization, which not only standardizes signal amplitudes across subjects but also preserves interregional signal differences more effectively than Pearson correlation. Furthermore, the proposed method incorporates cross-entropy techniques, offering an advanced perspective on the temporal ordering of signals between brain regions rather than merely capturing their synchronization. Experimental results demonstrate that the proposed subject-normalized cross-joint entropy achieves superior classification accuracy in schizophrenia, mild cognitive impairment, and autism spectrum disorder, outperforming the conventional normalized correlation method by approximately 4%, 6%, and 7%, respectively. Additionally, the observed performance improvement may be attributed to changes in the symmetry of functional connectivity between brain regions—an aspect often overlooked in traditional functional connectivity analyses.

## Highlights

• Propose a brain connectivity method based on subject normalization
• Limitations of cross-entropy supplementary correlation method
• Assesses the potential of cross-entropy in brain disease classification tasks
• Changes in the symmetry of brain entropy connectivity

## Graphical abstract

**Figure:**
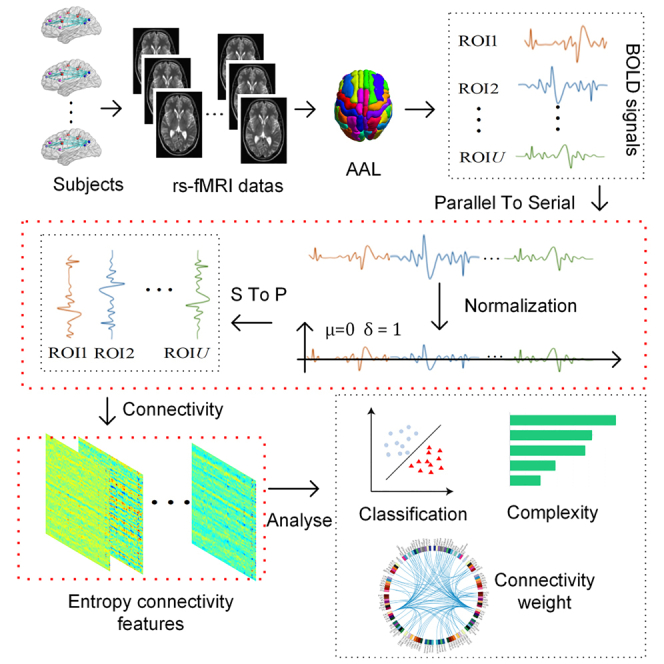

## Introduction

Brain connectivity, as delineated in previous research,^1^ encompasses the coordinated activity patterns among distinct brain regions either during task performance or during resting states, thereby unveiling temporal synchronicity among these regions. Within the field of neuroscience, particularly in the realm of brain network theory, numerous investigations have substantiated the pervasiveness of such connectivity. Brain network theory posits that neural elements within the brain are interconnected through intricate network architectures, giving rise to functional networks capable of dynamically modulating their connectivity patterns during cognitive engagements or states of rest. A prototypical example of such a functional network is the default mode network (DMN),^2^ whose activity of the DMN is purportedly least pronounced during cognitive tasks and most pronounced during resting state.^3^ The term “resting state” denotes an individual’s conscious state devoid of specific tasks or external stimuli, during which the brain remains notably active, fostering interregional interactions. Research underscores the pivotal role of the resting state in brain function, encompassing contributions to cognitive regulation, introspective processes, and self-referential cognition.^4^

Since brain connectivity serves as fundamental constituents of brain networks, they constitute the cornerstone for comprehending their essence. Typically, brain networks of individuals afflicted with brain disorders manifest notable disparities when juxtaposed with those of healthy counterparts. The distinctions are statistically discernible in the inter-regional connections within the brain, thereby offering prospects for adjunctive diagnosis of brain pathologies. Various neuroimaging modalities have been employed by researchers to delineate brain region connectivity, including positron emission tomography (PET),^5^ electroencephalography (EEG),^6^ magnetoencephalography (MEG),^7^ and functional magnetic resonance imaging (fMRI).^8^ Notably, resting-state fMRI (rs-fMRI) has emerged as a pivotal instrument for investigating brain connectivity, owing to its inherent advantages such as task-free acquisition, absence of ionizing radiation, and high spatial resolution.

When utilizing rs-fMRI data for the construction of brain connections, researchers typically extract blood-oxygen-level-dependent (BOLD) signals, from regions of interest (ROIs) and compute the Pearson correlation coefficient between them to quantify inter-brain connectivity. The Pearson correlation coefficient assesses the synchronicity of signals, representing the degree of similarity between two signal waveforms over time. In addition to the Pearson correlation coefficient, methodologies such as effective connectivity (EC)^9^^,^^10^^,^^11^ and dynamic causal modeling (DCM)^12^^,^^13^^,^^14^ are employed for brain area connectivity analysis. In recent years, entropy analysis,^11^^,^^15^ as an emerging approach in brain signal analysis, has gained traction in rs-fMRI studies. Particularly, the cross-entropy method delineates changes in information within brain regions by gauging the extent of signal disarray. Comparative to the Pearson correlation coefficient, entropy-based brain connectivity offers a novel perspective for discerning the independence and asynchrony among time series. The perspective is that, cross-entropy can differentiate between orthogonal and independent signals, thereby providing a valuable supplement to the limitations of the Pearson correlation coefficient. On the other hand, the Pearson correlation coefficient is used to determine whether two signals are synchronous. If the signals are not synchronized, further analysis becomes limited.

While the utilization of entropy methods in brain connectivity research is on the rise, current studies predominantly emphasize single entropy analyses,^16^^,^^17^^,^^18^^,^^19^^,^^20^ and the potential benefits of employing cross-entropy^21^^,^^22^^,^^23^^,^^24^^,^^25^ in the context of brain connectivity remain inadequately explored. Research has substantiated the distinctive impact of employing entropy-based connectivity methods in disease classification,^26^ yet a comprehensive assessment of cross-entropy’s efficacy in constructing brain networks is still lacking. Moreover, a myriad of approaches exist for implementing cross-entropy, encompassing information entropy, fuzzy entropy, Kolmogorov entropy, permutation entropy, and other single entropy calculation methodologies,^27^ alongside joint entropy, conditional entropy, divergence, and other cross-entropy calculation techniques.^28^^,^^29^ Hence, the exploration of optimal entropy calculation methods, including various forms of cross-entropy, holds paramount importance for informing and enhancing brain disease classification endeavors.

Regardless of whether employing Pearson correlation or entropy methods, normalizing BOLD time series constitutes a crucial step in constructing brain area connectivities. Normalization serves to standardize signals to a uniform scale and mitigate the adverse impact of outliers on time series computations. One commonly utilized normalization technique is the *Z* score method,^30^ which standardizes signals to a mean of 0 and a variance of 1. For instance, the Pearson correlation coefficient can be interpreted as the correlation between signals subsequent to *Z* score normalization. Additionally, linear normalization^31^ represents another prevalent normalization approach. For example, in the calculation of information entropy for discrete signals, scaling the signal to the interval between 0 and 1 facilitates the computation of signal probability distribution. However, both *Z* score normalization and linear normalization inevitably eliminate amplitude differences inherent in the brain region’s own signals, which may sometimes encapsulate group-specific information differences. Specifically, after preprocessing, the signal values from all ROIs can be obtained for each subject. Since the same brain atlas is used for extraction, the location of each ROI remains consistent across subjects. Applying *Z* score normalization to the ROIs ensures that signals from the same ROI in different subjects can be compared on a standardized scale. However, it is important to note that the amplitude of the signals from the same ROI may still vary across subjects.

To address the aforementioned challenges, this study introduces a brain entropy connectivity method grounded in subject normalization, with the objective of enhancing the investigation into classification disparities between brain disease and normal controls (NC) group. The contributions of this research are as follows. Firstly, to our best knowledge, the subject normalization method proposed in this paper has not been reported in the existing literature. Its advantage lies in its ability to not only address signal outliers but also preserve the amplitude information of each ROI region effectively. Secondly, the classification algorithm in this paper combines the new normalization method with an existing cross-entropy approach. For classifying neurological disorders, this new combination demonstrates better overall performance compared to the combination of existing normalization and connectivity algorithms. Finally, utilizing the proposed combination, strong symmetry in function connectivity was observed in brain disease, which may be a contributing factor to the improved classification performance.

In our experiment, we conducted classification tasks involving mild cognitive impairment (MCI), schizophrenia (SCZ), and autism spectrum disorder (ASD) alongside a control group. The three types of diseases are all believed to be closely related to abnormalities in both functional and structural connections of brain networks, and many researchers^3^^,^^15^^,^^17^^,^^19^^,^^26^ have already made significant contributions in this area. To ensure experiment reproducibility, fMRI data for the aforementioned disorders were sourced from publicly available databases. Evaluated entropy calculation methods encompass information entropy,^32^ conditional entropy,^28^ approximate entropy,^18^^,^^33^^,^^34^ sample entropy,^19^^,^^35^^,^^36^ fuzzy entropy,^20^^,^^37^ and permutation entropy,^38^ in addition to distribution entropy,^39^ spectral entropy,^40^^,^^41^ and Kolmogorov entropy.^42^ Cross-entropy methodologies include mutual information entropy,^43^^,^^44^ cross-conditional entropy,^22^^,^^45^ cross-approximate entropy,^21^^,^^46^^,^^47^ cross-sample entropy,^23^^,^^24^^,^^48^^,^^49^ cross-fuzzy entropy,^25^^,^^50^ cross-permutation entropy,^51^ cross-distribution entropy,^52^ cross-spectral entropy,^53^ and cross-Kolmogorov entropy.^53^ Experimental findings indicate that the proposed subject-normalized cross-joint entropy (CJE) yields superior classification accuracy. Under identical conditions, CJE outperforms Pearson correlation in the classification of SCZ, MCI, and ASD by approximately 4%, 6%, and 7%, respectively, representing a 7%, 12%, and 5% improvement over traditional normalized entropy methods, correspondingly.

## Results

### Classification performance

This section presents the classification outcomes obtained by integrating normalization with cross-entropy and correlation across three brain disease databases. Given the asymmetry of the cross-approximate entropy and cross-conditional entropy methods (wherein the entropy of the m-th and n-th ROIs is not equivalent to the entropy of the n-th and m-th ROIs), fully connected features are employed for classification. Additionally, the upper triangular feature classification of the entropy connection matrix is adopted for other methods. The subsequent analysis will delve into the classification performance of the proposed method within each database. To ensure the reliability of the results, we included the classification outcomes of K-Nearest Neighbors(KNN) and random forest (Figures S1 and S2). Additionally, we provided the confusion matrices, precision-recall rates (Tables S1–S3), and the receiver operating characteristic (ROC) curves (Figures S3–S5) for all three classifiers.

**Figure 1:**
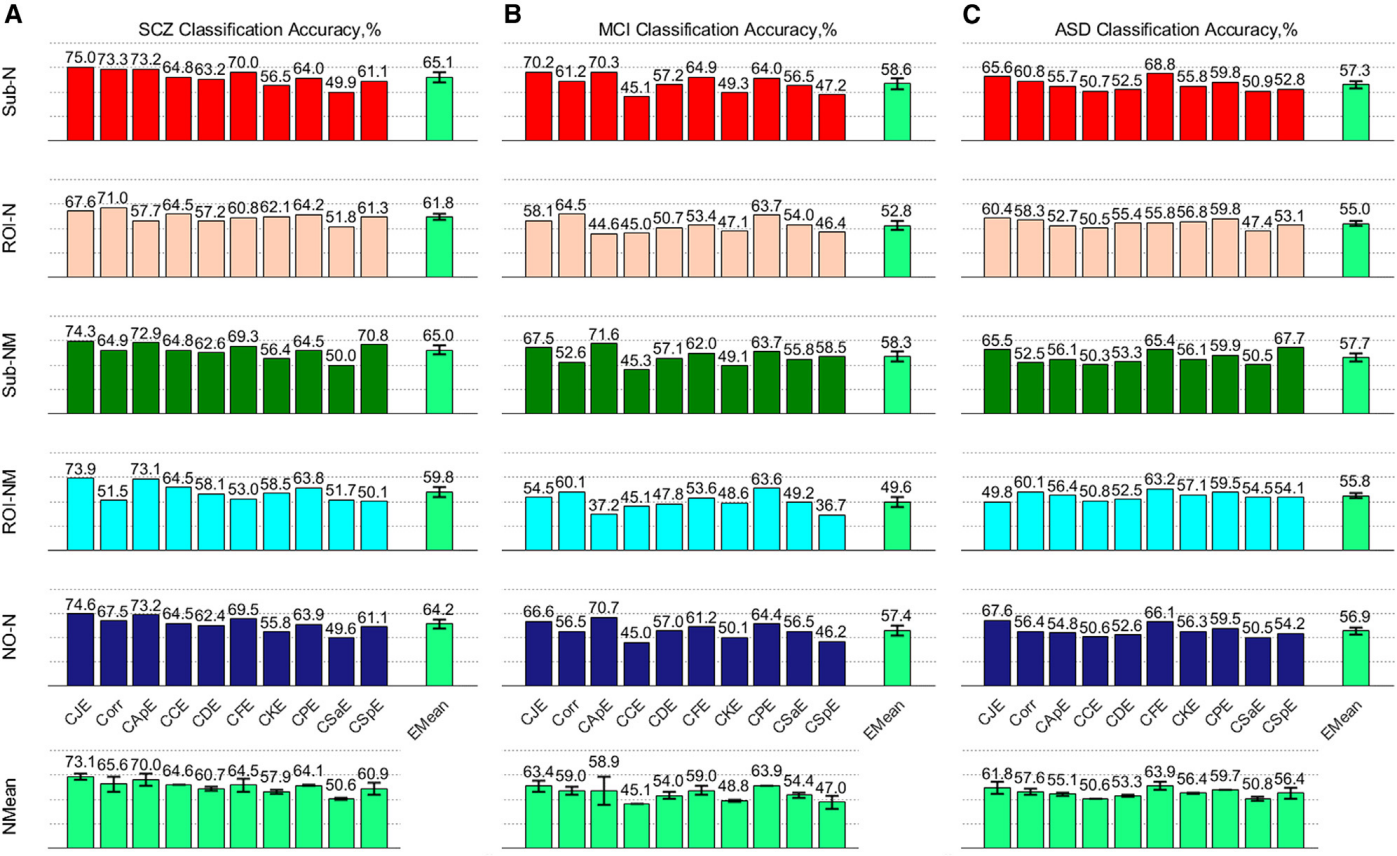
The average classification accuracy of each normalized cross-entropy (including correlation) across the three diseases of SCZ, MCI, and ASD is determined by combining 5 normalization methods and 10 cross-entropy measures, respectively (A) SCZ classification accuracy. (B) MCI classification accuracy. (C) ASD classification accuracy.

Figure 1 depicts the classification outcomes resulting from various combinations of normalization and cross-entropy/correlation methods. Furthermore, the mean value of cross-entropy within each normalization method (NMean) and the mean value of normalization within each cross-entropy measure (EMean) are also provided. In SCZ (see Figure 1A), the approach combining Sub_N and CJE yielded the highest classification accuracy of 75.0%. Notably, for the CJE cross-entropy method, it surpassed other cross-entropy and correlation methods across the remaining four normalization methods (apart from ROI_N). Additionally, for the Sub_N normalization method, it attained the highest classification accuracy among the six brain connection methods: CJE, Corr, CApE, CCE, CDE, and CFE. The average classification accuracy of Sub_N in each cross-entropy measure exceeded that of other normalization methods, and similarly, the average classification accuracy of CJE within each normalization method surpassed that of other entropy methods.

In MCI (see Figure 1B), the approach combining Sub_N and CJE attained a notable classification accuracy of 70.2%. Regarding the CJE method, except for ROI_N and ROI_NM, it secured the second-highest classification accuracy among the remaining three normalization methods. Furthermore, for the Sub_N normalization method, it achieved the first or second-highest classification accuracy in CJE, Corr, CDE, CFE, CPE, and CSaE. The average classification accuracy of Sub_N in each cross-entropy measure surpassed that of other normalization methods, while the average classification accuracy of CJE in each normalization method ranked second, closely following the CPE method.

In ASD (see Figure 1C), the approach combining Sub_N and CJE also yielded a notable classification accuracy of 65.6%. Regarding the CJE method, except for ROI_NM, it did not achieve the first or second-highest classification accuracy among the other four normalization methods. However, for the Sub_N normalization method, it achieved the first or second-highest classification accuracy in CJE, Corr, CCE, CFE, and CPE. The average classification accuracy of Sub_N in each cross-entropy measure closely followed Sub_NM, ranking second. Similarly, the average classification accuracy of CJE in each normalization method closely matched the CFE method, also ranking second.

### Computational complexity

**Figure 2:**
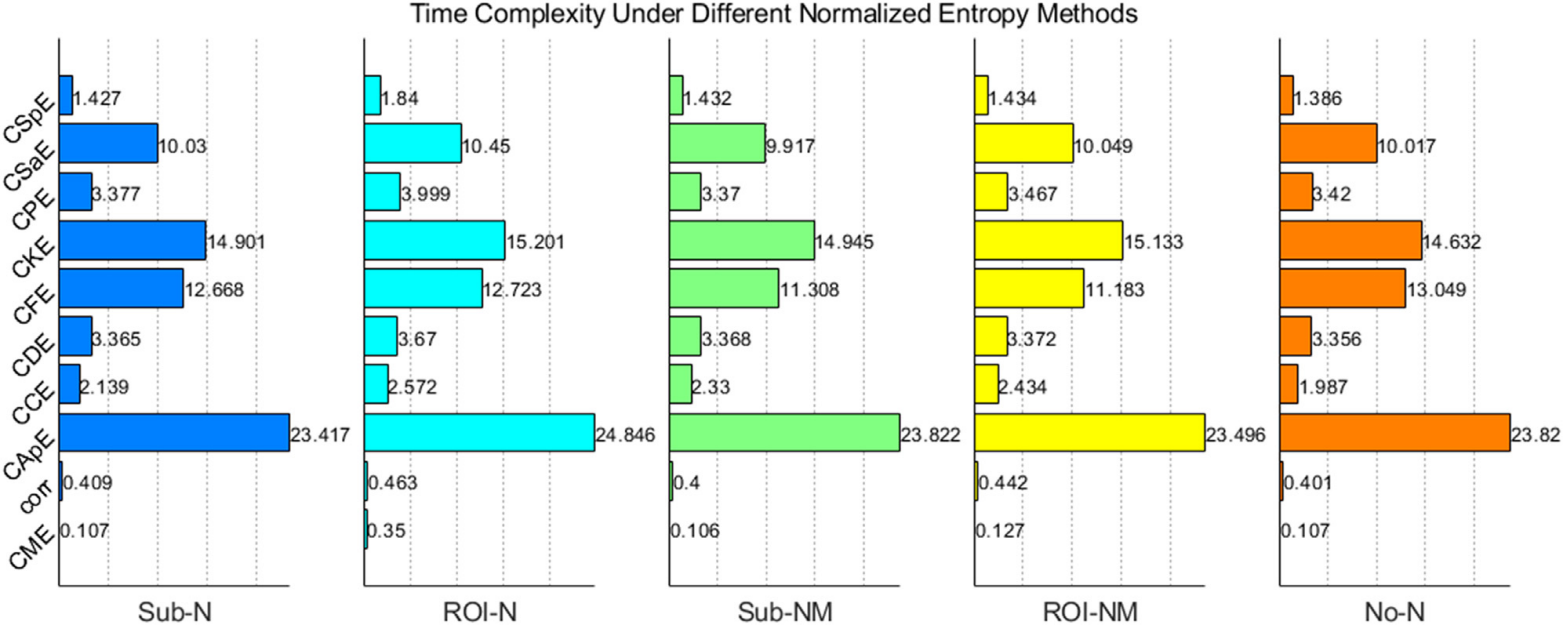
Histogram depicting the average running times (unit:s) of different normalized cross-entropy methods obtained across three pathologies

To assess the computational complexity of different methods, this section presents the running time of each cross-entropy/correlation method combined with normalization in Figure 2, averaged across the three databases. To ensure fairness, all connections in the matrix are calculated regardless of whether the upper and lower triangles of the connection matrix are symmetrical (cross-approximate entropy, cross-conditional entropy asymmetry). As depicted in Figure 2, for each normalization method, the calculation time does not vary significantly regardless of the brain connection calculation method used, although the calculation time of ROI_N is slightly longer. Furthermore, for each connection method, the calculation time of CJE is the shortest, even less than that of the Corr method.

### Weight analysis of entropy connectivities

**Figure 3:**
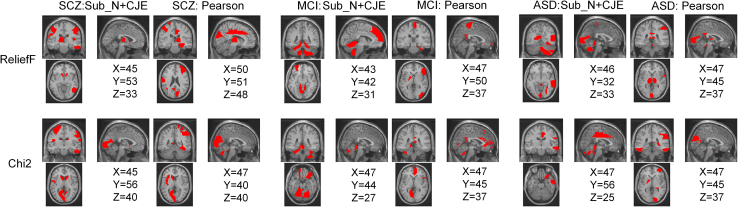
Within ASD, MCI, and SCZ, the ROIs with high-weighted connectivities to PCC are shown

The posterior cingulate cortex (PCC) is regarded as one of the central nodes of the entire brain during the resting state.^54^ We employed the ReliefF^55^ and Chi-square (Chi2)^56^ methods to compute the relationship between the PCC and various crucial brain regions. The connectivity weight primarily focuses on the results of Sub_N+CJE and ROI_N + Corr (i.e., Pearson correlation). Figure 3 illustrates the brain regions with high weights between ASD, MCI, SCZ, and the PCC, where the ROIs are derived from the AAL116 template. The high-weighted ROIs are the top 10 ROIs with the highest weights selected from all, denoted in red. The weights are determined through calculations using the ReliefF^55^ and Chi-square (Chi2)^56^ methods. The coordinates X, Y, and Z correspond to the Montreal Neurological Institute(MNI)space. It is evident from the figure that regardless of the method used to calculate the weights, the brain regions identified by CJE differ from those associated with Pearson correlation. For instance, considering ASD, the identical brain regions obtained by both methods include Cerebellum_4–5 and Cerebellum_6 in the left hemisphere and Cerebellum_8 in the right hemisphere, while the remaining regions differ.

**Figure 4:**
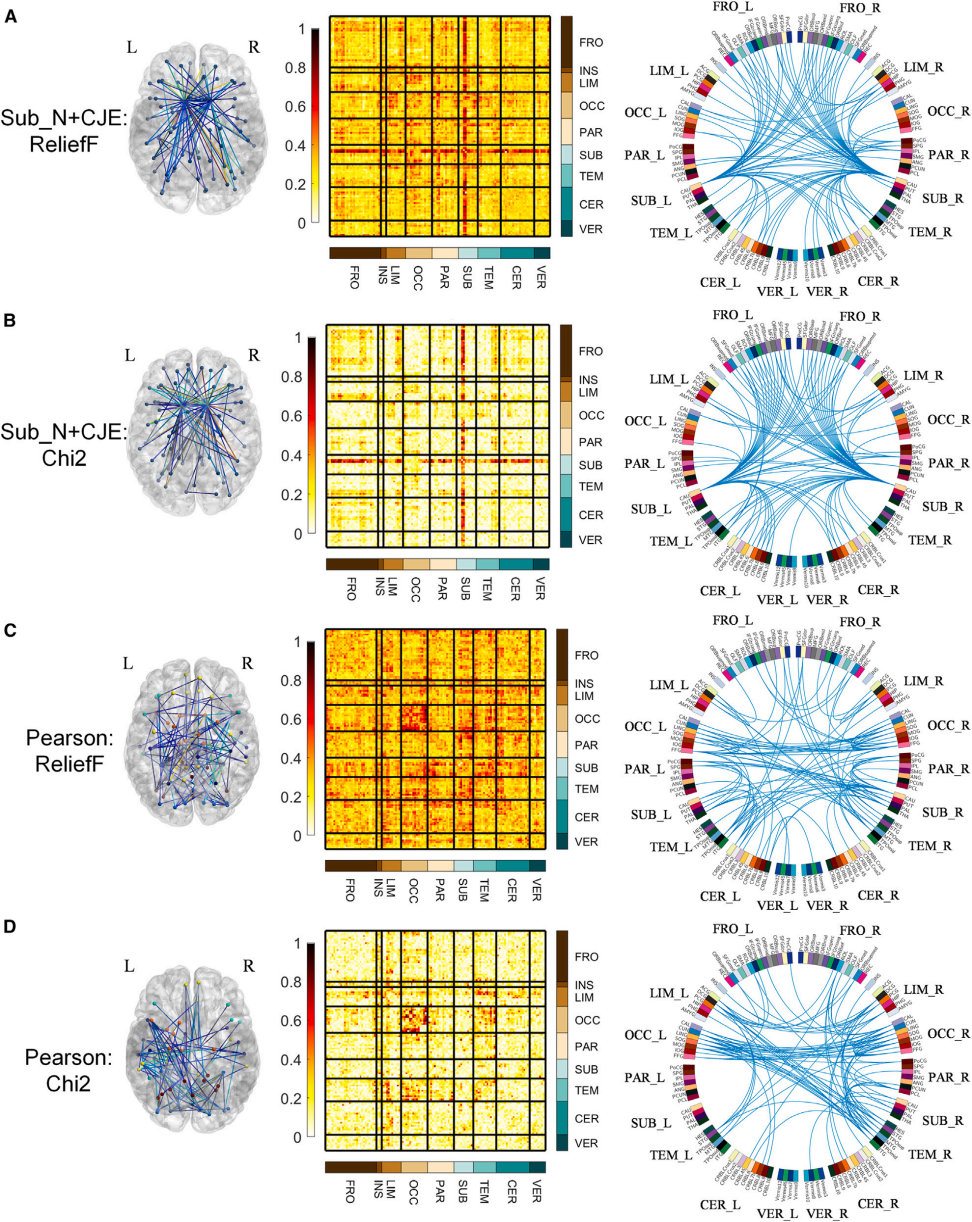
Sub_N+CJE and Pearson connectivity weights in SCZ (A) SCZ Connection weights calculated by ReliefF under Sub_N+CJE method. (B) SCZ Connection weights calculated by Chi2 under Sub_N+CJE method. (C) SCZ Connection weights calculated by ReliefF under Pearson correlation method. (D) SCZ Connection weights calculated by Chi2 under Pearson correlation method.

Figure 4 illustrates the Sub_N+CJE and ROI_N + Corr (i.e., Pearson correlation) connectivity weight diagram in SCZ, where the ROIs are from AAL116 and weights are calculated from ReliefF and Chi2. In the first column, the positions of the connectivities with the top 100 weights in the brain are represented, as visualized by BrainNet Viewer (https://www.nitrc.org/projects/bnv/); the second column displays the entropy connectivity weight matrix, where weight values have been normalized to range between 0 and 1, and rows and columns are arranged according to brain regions, including frontal lobe (FRO), insula (INS), limbic (LIM), occipital lobe (OCC), parietal lobe (PAR), subcortical (SUB), temporal lobe (TEM), cerebellum (CER), and vermis volume (VER); the third column illustrates the connectivity diagram with the top 100 weights, depicted using Circos (http://www.circos.ca/software/). The brain regions in the diagram are further divided into left and right hemispheres. In Figures (A) and (B), the brain connectivities are established using Sub_N+CJE, and the weight results obtained from relief and Chi2 methods exhibit similarity. High-weighted ROI connectivities are predominantly concentrated in the SUB area. Specifically, it is concentrated in the entropy connectivity between the caudate nucleus and other ROIs, and expresses partial symmetry in the left and right hemispheres. In Figures (C) and (D), the brain connectivities are established using Pearson correlation. The weight calculation results from both relief and Chi2 methods do not show the band-shaped area depicted in the second column of (A) and (B), and there is also a lack of symmetry in the circular connection diagram in the third column.

**Figure 5:**
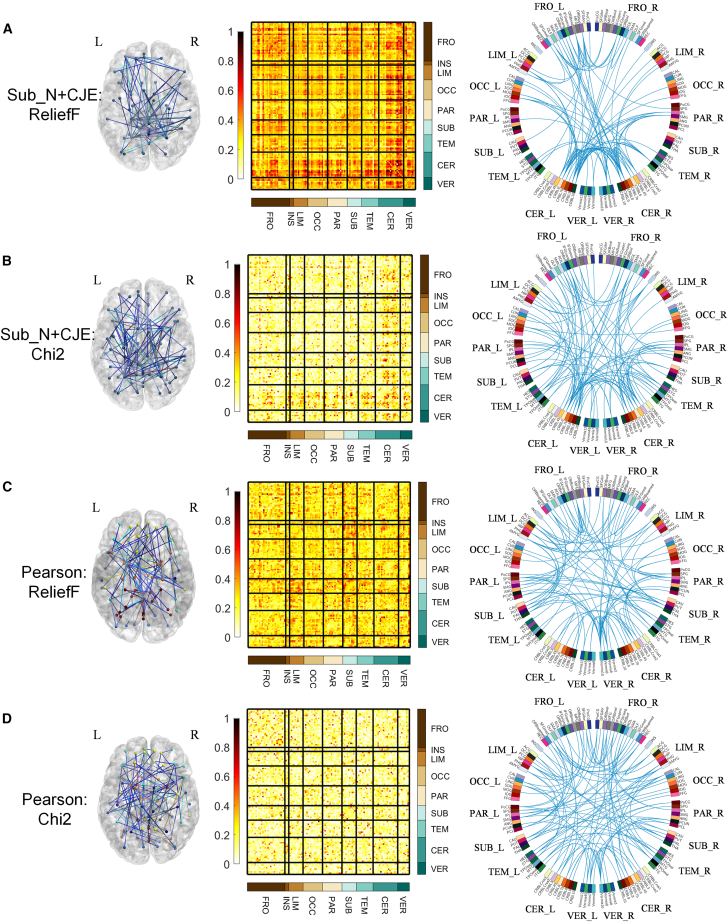
Sub_N+CJE and Pearson connectivity weights in MCI (A) MCI Connection weights calculated by ReliefF under Sub_N+CJE method. (B) MCI Connection weights calculated by Chi2 under Sub_N+CJE method. (C) MCI Connection weights calculated by ReliefF under Pearson correlation method. (D) MCI Connection weights calculated by Chi2 under Pearson correlation method.

Figure 5 illustrates the Sub_N+CJE and Pearson correlation connectivity weight diagram in MCI. In Figures (A) and (B), the connectivities established by Sub_N+CJE also exhibit similarities in the two weight calculation results of ReliefF and Chi2. High-weighted ROI connectivities are predominantly concentrated in the cerebellum (CER) area. Conversely, the Pearson correlation method (Figures 5C and 5D) did not identify brain regions with significantly high weights.

**Figure 6:**
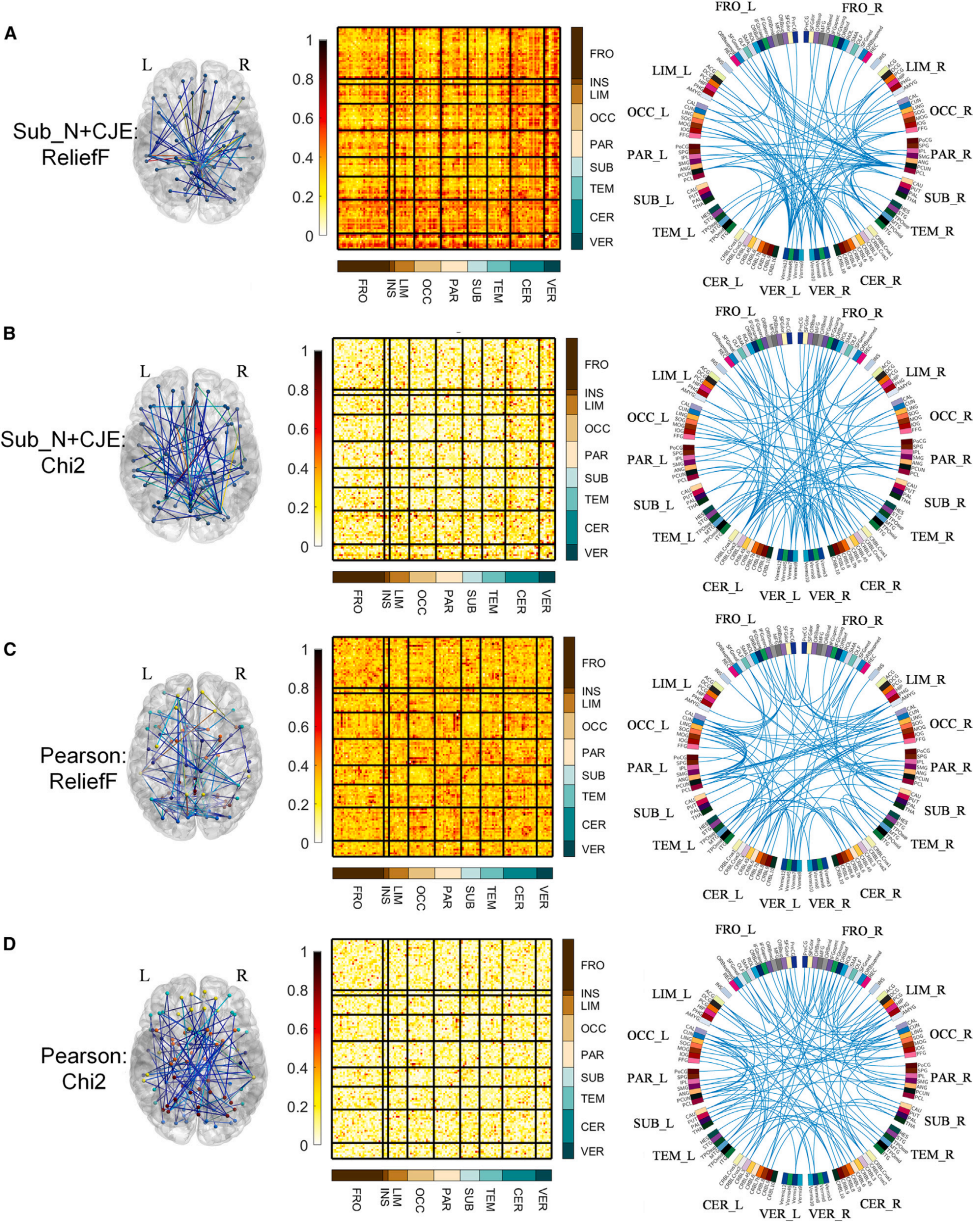
Sub_N+CJE and Pearson connectivity weights in ASD (A) ASD Connection weights calculated by ReliefF under Sub_N+CJE method. (B) ASD Connection weights calculated by Chi2 under Sub_N+CJE method. (C) ASD Connection weights calculated by ReliefF under Pearson correlation method. (D) ASD Connection weights calculated by Chi2 under Pearson correlation method.

Figure 6 illustrates the Sub_N+CJE and Pearson correlation connectivity weight diagram in ASD. Similar to the findings in Figures 4 and 5, the high-weighted connections in Figures (A) and (B) exhibit more pronounced regional characteristics, with higher weights observed in the ROIs connected to the CER and VER. Conversely, the high-weighted ROIs identified by Pearson correlation in Figures (C) and (D) are more evenly distributed.

### Symmetry analysis of entropy connectivities

**Figure 7:**
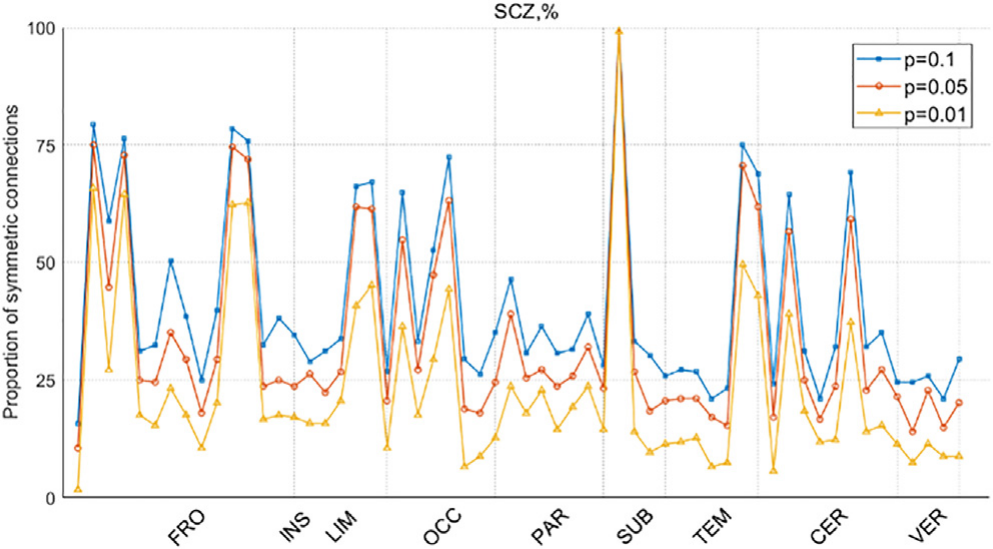
The proportion of ROI symmetric connectivities is presented for Sub_N+CJE across different significance levels in SCZ

**Figure 8:**
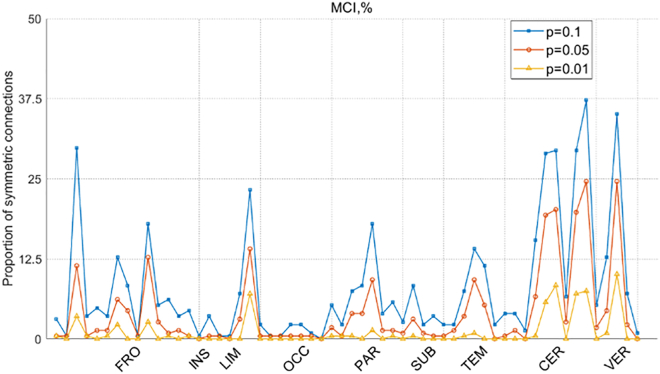
The proportion of ROI symmetric connectivities is presented for Sub_N+CJE across different significance levels in MCI

**Figure 9:**
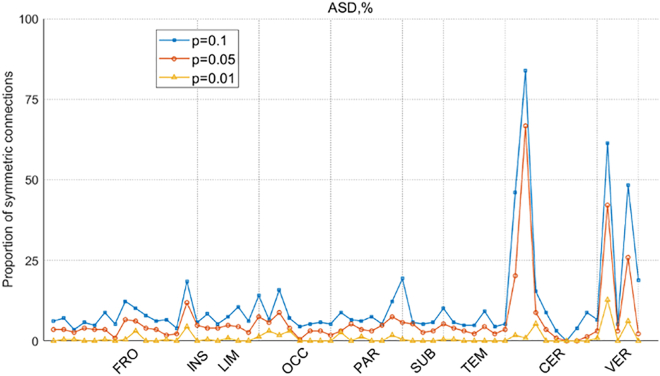
The proportion of ROI symmetric connectivities is presented for Sub_N+CJE across different significance levels in ASD

Note that the functional connectivity results obtained using the methods outlined in the previous section revealed strong symmetry in certain brain regions, a pattern rarely observed in prior functional connectivity analyses. Therefore, this section presents additional experimental results to further characterize this symmetry. The proportion of symmetric entropy connections in the three diseases is calculated. Figure 7 illustrates the proportion of symmetric connections in the ROI nodes of Sub_N+CJE in SCZ, the horizontal axis represents the brain regions corresponding to the 116 ROIs from the automatic anatomical labeling (AAL) brain atlas, while the vertical axis indicates the proportion of symmetric connectivities relative to all connections for each ROI node. The detailed correspondence between ROI and brain region can be found in Table S4 of the supplementary information. As shown in the figure, several brain regions in SCZ exhibit nodes with high symmetry under three different p values. Notably, the caudate nucleus node in the SUB brain region has the highest proportion of symmetric connections, approximately 99%, across all p values, while nodes in other brain regions have proportions below 80%. Figure 8 presents the symmetry proportions of ROI connections for Sub_N+CJE nodes in MCI, where the CER and VER brain regions, both belonging to the cerebellum, have the greatest number of nodes with higher symmetry proportions. Figure 9 depicts the proportion of symmetric connections in the ROI nodes of Sub_N+CJE in ASD. In this case, nodes with higher symmetry are also found in the CER and VER regions of the cerebellum, with the proportion of symmetric connections in other brain regions being less than 25% across the three p values.

### Graph theory analysis of entropy connectivity

In this subsection, the Sub_N+CJE method is applied to construct brain connectivity maps for three diseases: SCZ, MCI, and ASD. Initially, ReliefF is utilized to compute the weight of each entropy connectivity. The entropy connectivities with the top 100 weights are then selected and normalized between 0 and 1. Subsequently, connectivities with entropy values below a threshold are retained to form the graph network, where the threshold is set to 1/3. The graph features listed in experimental methods and classification section are extracted from the network. Following feature extraction, a two-sample t test is conducted with a significance level of p=0.05. Table 1 shows the results of SCZ. Among its 7 features, except global efficiency and node strength, which showed results of p< 0.05, the other features were all p< 0.01. Table 2 shows the results of MCI, in which global efficiency, node strength and participation coefficient showed no difference, p>0.05, and the remaining four features all showed p<0.05. Table 3 shows the results of ASD, and its 7 features all show differences at the p<0.05 level.

**Table 1:** Graph features of brain connectivity network constructed by Sub_N+CJE in SCZ

Features	Group (mean ± standard deviation)		*t* value	*p* value
	SCZ	NC		
Global efficiency	0.0226 ± 0.0165	0.0286 ± 0.0161	−2.2304	<0.05∗
Modular	0.3207 ± 0.1442	0.1967 ± 0.0719	6.5101	<0.01∗∗
Isogamy	−0.6368 ± 0.1859	−0.7868 ± 0.0731	6.3438	<0.01∗∗
Average traffic	0.0420 ± 0.0284	0.0233 ± 0.0123	5.1235	<0.01∗∗
Node strength	0.5605 ± 0.2399	0.6489 ± 0.2373	−2.2306	<0.05∗
Clustering coefficient	0.0635 ± 0.0505	0.0972 ± 0.0540	−3.8771	<0.01∗∗
Participation coefficient	0.0434 ± 0.0285	0.0598 ± 0.0292	−3.4069	<0.01∗∗

Note: ∗indicates p<0.05, ∗∗indicates p<0.01

**Table 2:** Graph features of brain connectivity network constructed by Sub_N+CJE in MCI

Features	Group (mean ± standard deviation)		*t* value	*p* value
	MCI	NC		
Global efficiency	0.0184 ± 0.0113	0.0214 ± 0.0146	−0.9127	0.36
Modular	0.5110 ± 0.0701	0.4140 ± 0.1088	4.2399	<0.01∗∗
Isogamy	−0.4763 ± 0.1346	−0.5479 ± 0.1417	2.0724	<0.05∗
Average traffic	0.0606 ± 0.0225	0.0397 ± 0.0185	4.0635	<0.01∗∗
Node strength	0.5350 ± 0.2192	0.5636 ± 0.2926	−0.4418	0.66
Clustering coefficient	0.0321 ± 0.0255	0.0465 ± 0.0319	−2.0001	<0.05∗
Participation coefficient	0.0268 ± 0.0186	0.0350 ± 0.0243	−1.5161	0.13

**Table 3:** Graph features of brain connectivity network constructed by Sub_N+CJE in ASD

Features	Group (mean ± standard deviation)		*t* value	*p* value
	ASD	NC		
Global efficiency	0.0121 ± 0.0101	0.0241 ± 0.0144	−4.7844	<0.01∗∗
Modular	0.6182 ± 0.1070	0.5601 ± 0.1367	2.3379	<0.05∗
Isogamy	−0.4022 ± 0.1294	−0.3340 ± 0.1506	−2.4003	<0.05∗
Average traffic	0.0681 ± 0.0338	0.0955 ± 0.0345	−3.9752	<0.01∗∗
Node strength	0.3948 ± 0.1840	0.5841 ± 0.2363	−4.4139	<0.01∗∗
Clustering coefficient	0.0093 ± 0.0163	0.0262 ± 0.0272	−3.7521	<0.01∗∗
Participation coefficient	0.0176 ± 0.0139	0.0329 ± 0.0174	−4.7950	<0.01∗∗

## Discussion

This paper utilizes rs-fMRI data to analyze brain region connectivity, with the goal of distinguishing between disease and healthy control groups. While signal correlation-based methods, such as the Pearson correlation coefficient, are widely used for quantifying inter-node connectivity, entropy-based methods have gained traction in recent years due to their ability to address some limitations of correlation methods. However, regardless of the connectivity analysis method employed, preprocessing of raw node signals is essential. Traditional ROI-based normalization methods may overlook crucial amplitude information, which could be pivotal in elucidating group differences. Moreover, while some studies have explored entropy-based methods across various diseases, systematic comparative analyses are lacking. Therefore, this study focuses on exploring an effective cross-entropy calculation method to uncover commonalities and differences across different disease contexts. We employed subject normalization combined with ten entropy-based brain region connectivity methods to compute and analyze data from three diseases, aiming to identify reliable cross-entropy features to enhance classification accuracy and explore variations between brain regions. Through this approach, we aim to offer new insights into the intricacies of brain function and its implications in disease.

In traditional Pearson correlation analysis, the impact of signal amplitude is often disregarded. This method typically standardizes the mean of two signals to 0 and the variance to 1, assessing connectivity on a standardized scale. However, variations in signal amplitude exist across different brain regions due to task engagement or spontaneous activity during the resting state, which may be altered in brain diseases. To address this, our study proposes a subject normalization method that preserves relative signal magnitude when evaluating brain region connectivity changes. Compared to traditional methods, it offers greater signal differentiation and potential for enhanced classification accuracy.

Additionally, the Pearson correlation coefficient, while commonly used, has limitations in capturing signal relationships. It may overlook similarity between signals, potentially leading to inaccurate assessments of connectivity. To address this issue, our study employs the cross-entropy method. For example, when computing cross-joint entropy between signals, a smaller entropy value between a sinusoidal signal and a cosine signal indicates the relevance of signal relationships, instead of mere a phase difference. Moreover, the Pearson correlation coefficient can assess whether two signals are synchronized, but it cannot provide further insight when the signals are not synchronized. In contrast, cross-joint entropy accounts for both synchronization and independence. When the signals are synchronized, the entropy is lower due to reduced uncertainty. When the signals are not synchronized, they may still exhibit orthogonality, meaning they are uncorrelated but not entirely independent. In such cases, while the entropy is relatively high, it does not reach its maximum. If the signals are fully independent, there is no form of dependence or shared information between them, resulting in the maximum entropy value.

During implementation, we applied five different normalization techniques to rs-fMRI data, each preserving varying degrees of activity differences and influencing outcomes differently. To overcome limitations of traditional correlation approaches, we utilized various cross-entropy methods to establish brain region connectivity and employed the features for classification.

### Comparison of normalization and cross-entropy

The experimental findings presented in this article underscore the effectiveness of subject normalization methods, particularly subject *Z* score normalization and subject linear normalization, in achieving the highest classification accuracy among the three diseases studied. Conversely, the classification results of ROI normalization techniques, such as ROI *Z* score normalization and ROI linear normalization, are comparatively lower, and in some cases, even lower than the unnormalized results. For example, when employing the cross-approximate entropy method in MCI, the classification outcomes obtained without normalization notably surpass those obtained with ROI normalization. This indicates that valuable information inherent in the original signal, such as amplitude information, contributes to classification accuracy but is lost during ROI normalization. In contrast, subject normalization methods retain this crucial information. Furthermore, subject *Z* score normalization emerges as superior to subject linear normalization, achieving the highest mean classification accuracy in SCZ and MCI, surpassing other normalization methods. While it ranks second in ASD, its performance remains comparable to that of subject linear normalization. Overall, in comparison to existing combination algorithms, the new method proposed in this paper ranks among the top three in terms of classification accuracy for the three diseases, whereas the existing algorithms rank lower. Although some combination algorithms achieve high rankings, this is primarily due to the integration of the existing cross-entropy method with the subject normalization approach introduced in this paper, further demonstrating the superiority of the subject normalization.

The concept of cross-entropy, elaborated upon in Table 4 and the “Related Works on Entropy” section of this paper, serves as a valuable tool for analyzing information interaction in brain connectivity. While single entropy is effective at analyzing disordered changes in time series, it lacks the capacity to express alterations in brain area connections. Hence, cross-entropy is proposed to examine the brain’s information interaction from a connectivity or correlation perspective. The diverse cross-entropy classification accuracy rates presented in this study illustrate that the cross-joint entropy method outperforms other methods in classifying the three diseases. Specifically, in SCZ, cross-joint entropy achieves the highest mean classification accuracy, ranking first; in MCI, it ranks second, similar to the first cross-permutation entropy method; in ASD, it also ranks second, marginally lower than the first cross-fuzzy entropy by 2.1%. However, cross-permutation entropy and cross-fuzzy entropy do not exhibit higher classification accuracy in other diseases. Furthermore, in terms of average running time, cross-joint entropy demonstrates significantly faster operation compared to other cross-entropy methods or related approaches, ensuring higher operational efficiency.

**Table 4:** Common single entropy and cross-entropy

Type	Name	Expression
Single entropy	Information entropy	$H_{I}\left(u\right)=-\sum_{m}p\left(u\left(m\right)\right)\log\left(p\left(u\left(m\right)\right)\right)$, where u(m) is the element of the time series vector u, m=1,2,… are the sampling points, and p(·) represents the probability
Single entropy	Conditional entropy^28^^,^^45^	$H_{c}\left(u\right)=-\sum_{d-1}p\left(u_{d-1}^{\prime }\right)\sum_{m|d-1}p\left(\frac{u\left(m\right)}{u_{d-1}^{\prime }}\right)\log\;p\left(\frac{u\left(m\right)}{u_{d-1}^{\prime }}\right)$, where $p\left(u_{d-1}^{\prime }\right)$ represents the joint probability of mode $u_{d-1}^{\prime }$ after d dimensional phase space reconstruction, and $p\left(\frac{u\left(m\right)}{u_{d-1}^{\prime }}\right)$ represents the probability of sample u(m) under a given mode $u_{d-1}^{\prime }$
Single entropy	Approximate entropy^34^	$H_{a}\left(u\right)=\phi^{d}\left(r\right)+\phi^{d+1}\left(r\right)$,where r represents the similarity tolerance threshold, $\phi^{d}\left(r\right)=\left(1/\left(T-d+1\right)\right)\sum_{i=1}^{T-d+1}\log\left(C_{i}^{d}\left(r\right)\right)$ , $C_{i}^{d}\left(r\right)$ is the approximate ratio, T is the number of data sampling points in the time series
Single entropy	Sample entropy^36^	$H_{\text{sa}}\left(u\right)={\ln\;\phi }^{\prime d}\left(r\right)-{\ln\;\phi }^{\prime d+1}\left(r\right)$,where $\phi^{\prime d}\left(r\right)=\left(1/\left(T-d+1\right)\right)\sum_{i=1}^{T-d}{C^{\prime }}_{i}^{d}\left(r\right)$, ${C^{\prime }}_{i}^{d}\left(r\right)=\text{num}\left(d^{\prime }\right)/\left(T-d\right)$, $d^{\prime }$ represents the distance between corresponding position elements between subsequences after reconstructing u
Single entropy	Fuzzy entropy^37^	$H_{F}\left(u\right)={\ln\;\phi }^{\prime \prime d}\left(\eta ,r^{\prime }\right)-{\ln\;\phi }^{\prime \prime d+1}\left(\eta ,r^{\prime }\right)$, where $\phi^{\prime \prime d}\left(\eta ,r^{\prime }\right)=\left(1/\left(T-d\right)\right)$$\sum_{i=1}^{T-d}\left(\frac{1}{T-d-1}\sum_{j=1,j\neq i}^{T-d}D_{ij}^{d}\left(\eta ,r^{\prime }\right)\right)$, $r^{\prime }$ is the width, $D_{ij}^{d}$ is the fuzzy membership degree, η is the gradient
Single entropy	Permutation entropy^38^	$H_{P}\left(u\right)=-\sum_{i=1}^{T-\left(d-1\right)\tau }p\left(i\right)\log\;p\left(i\right)$,where τ represents the delay time, and p(i) represents the probability of occurrence of the *i*-th arrangement in sequence u
Single entropy	Distribution entropy^39^	$H_{D}\left(u\right)=-\left(\frac{1}{\log\;M}\right)\sum_{m=1}^{M}p_{m}\;\log\left(p_{m}\right)$,where m is the m-th quantization interval, and M is the total number of quantization intervals
Single entropy	Spectral entropy^41^	$H_{\text{se}}\left(u\right)=-\log\;P\left(f_{m}\right)$, where $f_{m}$ is the m-th sample value on the power spectrum, and $P\left(f_{m}\right)$ represents the energy proportion of each frequency band
Single entropy	Kolmogorov entropy^42^	$K_{2}\left(u\right)=\lim_{r^{\prime \prime }\to 0}\lim_{d\to \infty }\frac{1}{\tau }\ln\left(\frac{C_{d}^{\prime \prime }\left(r^{\prime \prime }\right)}{C_{d+1}^{\prime \prime }\left(r^{\prime \prime }\right)}\right)$, where $r^{\prime \prime }$ represents the critical distance, $C_{d}^{\prime \prime }\left(r^{\prime \prime }\right)$ is the associated integral
Cross-entropy	Mutual information entropy^44^	$H_{I}\left(u,v\right)=H\left(u\right)+H\left(v\right)-H\left(u,v\right)$, where u and v represent two different time series vectors respectively, $H\left(u,v\right)=-\sum_{m=1}^{T}\sum_{n=1}^{T}p\left(u\left(m\right),v\left(n\right)\right)\log\;p\left(u\left(m\right),v\left(n\right)\right)$
Cross-entropy	Cross-conditional entropy^28^^,^^45^	$H_{\text{CCE}}\left(u,v\right)=-\sum_{d-1}p\left(v_{d-1}^{\prime }\right)\sum_{m|d-1}p\left(u\left(i\right)|v_{d-1}^{\prime }\right)\log\;p\left(u\left(i\right)|v_{d-1}^{\prime }\right)$
Cross-entropy	Cross-approximate entropy^46^	$H_{\text{CApE}}\left(u,v\right)=\phi_{ij}^{d}\left(r\right)-\phi_{ij}^{d+1}\left(r\right)$, where $\phi_{ij}^{d}\left(r\right)$ represents the degree of approximation between the i-th sequence segment of the time series vector $u^{\prime }$ reconstructed by u and v and the j-th sequence segment of $v^{\prime }$
Cross-entropy	Cross-sample entropy^48^^,^^49^	$H_{\text{CSaE}}\left(u,v\right)=-\ln\left(\frac{A^{d}\left(r\right)}{B^{d}\left(r\right)}\right)$,where $A^{d}\left(r\right)=\frac{\sum_{i=1}^{T-d}A_{i}^{d}\left(r\right)}{T-d}$, $A_{i}^{d}\left(r\right)$ represents the number of distances between corresponding samples of the reconstructed vectors $u^{\prime }$ and $v^{\prime }$ in the d+1 dimensional phase space that are less than or equal to the threshold r, Similarly, can be obtained $B^{d}\left(r\right)$, $B_{i}^{d}\left(r\right)$ is obtained from the d dimensional phase space
Cross-entropy	Cross-fuzzy entropy^50^	$H_{\text{CFE}}\left(u,v\right)={\ln\;\phi^{d}}^{\prime \prime }\left(r\right)-{\ln\;\phi^{d+1}}^{\prime \prime }\left(r\right)$, where ${\ln\;\phi^{d}}^{\prime \prime }\left(r\right)=\frac{1}{T-d}\sum_{i=1}^{T-d}\left(\frac{1}{N-d}\sum_{j=1}^{T-d}D_{ij}^{d}\right),D_{ij}^{d}$ represents the fuzzy similarity between $u_{i}^{\prime }$ and $v_{j}^{\prime }$
Cross- entropy	Cross-permutation entropy^51^	$H_{\text{CPE}}\left(u,v\right)=-\sum_{m=0}^{M}p\left(\delta_{m}\right)\log\;p\left(\delta_{m}\right)$, where $\delta_{m}$ represents the m-th arrangement of the reconstructed sequence from u and v
Cross-entropy	Cross-distribution entropy^52^	$H_{\text{CDE}}\left(u,v\right)=\frac{1}{\ln\left(a\right)}\frac{1}{q-1}\left(1-\sum_{m=1}^{M}p_{m}^{q}\right)$, where q is the Tsallis entropy order, a is the base, M is the number of intervals, and $p_{m}^{q}$ represents the probability of falling in the m-th interval
Cross-entropy	Cross-spectral entropy^53^	$H_{\text{CSpE}}\left(u,v\right)=-\sum_{m}P_{uv}\left(f_{m}\right)\log\;P_{uv}\left(f_{m}\right)$, where $f_{m}$ is the m-th sample value of the mutual power spectrum of u and v, and $P\left(f_{m}\right)$ is the energy proportion
Cross-entropy	Cross-Kolmogorov entropy^53^	According to the definition of simple entropy, the distance between phase points is calculated as $d_{ij}=\left|u^{\prime \left(i\right)}-v^{\prime \left(j\right)}\right|$ , substituted into the correlation integral, and then substituted into the Kolmogorov entropy formula to get

Based on the foregoing analysis, this paper advocates for the combination of subject *Z* score normalization and cross-joint entropy as a superior approach for constructing brain entropy connections. Experimental findings indicate that this combination achieves classification accuracies of 75.0% (ranked 1st out of 50 methods, 1/50), 70.2% (4/50), and 65.6% (5/50) in SCZ, MCI, and ASD diseases, respectively. Although these figures are slightly lower than those achieved by cross-approximate entropy and cross-spectral entropy with subject linear normalization in the MCI disease database and ASD, it is noteworthy that the methods did not consistently demonstrate good classification accuracy across all conditions. For instance, subject linearly normalized cross-approximate entropy achieved a classification accuracy of 71.6% in MCI but only 56.1% in ASD.

### Entropy connectivity analysis for brain diseases

In SCZ, our investigation into connectivities established through subject *Z* score normalization and cross-joint entropy revealed significant patterns highlighted by ReliefF and Chi-square tests. Notably, substantial disparities emerged in connectivities between the subcortical region and various cortical regions, such as the frontal lobe, limbic system, parietal lobe, and cerebellum. Particularly striking were connectivities involving the caudate nodes, which displayed substantial weighting and demonstrated robust symmetry between the left and right hemispheres. These findings align with previous research indicating structural disparities in the prefrontal lobe among individuals with schizophrenia, correlating with dysfunctions in cognitive control, decision-making, and emotion regulation.^57^ Moreover, the observed symmetrical high-weighted connections, especially those linking other cortical regions with the caudate nucleus, align with existing research on hemispheric specialization in schizophrenia patients. Specifically, findings suggest weakened left hemisphere specialization and strengthened right hemisphere specialization,^58^ indicative of connectivity disparities in both hemispheres of SCZ patients. The identified symmetry in caudate nucleus connections holds significance in elucidating SCZ pathology. Additionally, our study revealed that connections involving the caudate nucleus exhibited greater feature weights, supporting genetic inquiries implicating dopamine-related mechanisms in SCZ onset,^59^ with the caudate nucleus playing a pivotal role as a dopaminergic synapse distribution area.^60^ Notably, these insights were not gleaned through Pearson correlation analysis.

In our investigation of MCI, notable features emerged in connectivities established through the amalgamation of subject *Z* score normalization and cross-joint entropy, predominantly observed in 26 cerebellar nodes. Remarkably, similar patterns were evident when analyzing connectivities with the PCC nodes, highlighting the cerebellum’s pivotal role in cognitive functions. Traditionally recognized for its involvement in movement coordination, the cerebellum has often been sidelined in cognitive studies,^61^^,^^62^ possibly due to the misconception that ROI normalization diminishes differences in cerebellar nodes. However, mounting evidence underscores the cerebellum’s intricate involvement in cognitive and emotional processing.^63^ Functional MRI analyses suggest that the cerebellum regulates cognition and emotion similar to its role in motor control, presenting potential avenues for neuropsychiatric interventions. Additionally, our findings revealed an abundance of high-weighted connectivities within the internal connectivities of the frontal lobe, particularly prevalent in the left hemisphere. Previous research on MCI has emphasized enhanced functional connectivity in the frontal lobe among patients, notably in the left frontal lobe, which exhibits higher overall functional connectivity.^64^ Studies employing synchronized rhythmic brain stimulation have shown a preferential increase in frontal lobe activity following stimulation,^65^ leading to rapid improvements in working memory performance. Moreover, investigations into the topology of hemispheric white matter networks in Alzheimer’s disease (AD) and MCI patients have identified significant group disparities primarily in the left hemisphere,^66^ suggesting its crucial role in abnormal topological asymmetry among patients. These findings collectively deepen our understanding of brain connectivity disparities in MCI pathology.

In ASD, our analysis unveiled disparities in feature weights across the entire brain connectivity domain, signaling widespread connectivity alterations rather than isolated changes in specific brain regions. Particularly notable were the significant differences observed in the cerebellum, where connectivity exhibited higher weights predominantly, especially in connectivity with the PCC. While previous research has primarily concentrated on findings in the frontal and temporal lobes,^67^ attributing to their integral roles in high-level cognitive functions, such as those in the medial prefrontal cortex, posterior cingulate cortex, and subparietal cortex. Notably, global hypoconnectivity within lobules and sensorimotor regions, particularly involving the medial prefrontal cortex and posterior cingulate cortex, has been implicated in social impairment.^68^ Studies utilizing independent component analysis (ICA) and ROI-based seed points have reported underconnectivity in regions such as the insula (saliency network) and amygdala (medial temporal lobe network).^69^ Furthermore, investigations into the cerebellum have unearthed enhanced local connection patterns among adolescent ASD patients,^70^ further substantiating the connectivity abnormalities observed in our analysis. Moreover, studies leveraging graph theory to explore various rs-fMRI studies in ASD patients have highlighted instances of both over- and under-connectivity, emphasizing the complexity of whole-brain connectivity alterations in ASD and the challenge in identifying overarching trends or patterns of change.^71^ Recent research corroborated these findings by demonstrating widespread whole-brain functional alterations in ASD patients, with differences evident across nearly all brain regions.^72^ These observations align with our findings, suggesting that ASD patients exhibit a more dispersed pattern of brain network connectivity.^73^ Consequently, future research on ASD may benefit from a more comprehensive focus on understanding whole-brain connectivities and their implications for the disorder.

This study conducted a comprehensive statistical analysis of the impact of three brain disorders on the symmetry of brain connectivity patterns. In typical brain function, information exchange is facilitated by the coordination of multiple brain regions. When brain disorders occur, they disrupt this exchange, and these disruptions can be statistically observed as significant differences from normal control groups. If such differences manifest symmetrically in brain connectivity, it provides valuable insights into how brain disorders affect neural communication patterns. The findings of this study indicate that, in SCZ, symmetrical connectivity disruptions were observed in the frontal lobe, limbic system, occipital lobe, subcortical structures, temporal lobe, and cerebellum, with the highest statistical value occurring in the caudate nucleus within the subcortical structures. This suggests that all nodes connected to the caudate nucleus are affected by SCZ, consistent with the feature importance analysis, further reinforcing the hypothesis that the caudate nucleus may be a critical locus for understanding SCZ. In MCI, while the differences in connectivity symmetry between nodes were not pronounced, the majority of nodes with higher proportions of symmetrical connections were concentrated in the cerebellum. This highlights the involvement of the cerebellum in cognitive function and suggests that cerebellar function is compromised in MCI patients. In ASD, a similar pattern was observed, with a greater proportion of symmetrical connections concentrated in the cerebellum, while asymmetrical connectivity differences in other brain regions were minimal. The discovery of symmetrical connection patterns for the three disorders, identified through the *Z* score normalized association with CJE, exhibits significant disparities when compared to the Pearson correlation method. This implies that the approach presented in this paper makes a distinctive contribution to brain disease classification tasks.

To further elucidate the distinctions between disease and normal control groups in the connectivity established by subject *Z* score normalization and cross-joint entropy, we constructed corresponding graph representations and extracted features for statistical hypothesis testing. The results revealed that across SCZ, MCI, and ASD groups, global efficiency, node strength, clustering coefficient, and participation coefficient were all significantly lower compared to the NC group (p < 0.05). This suggests that the level of interactivity between nodes in the patient group was diminished relative to the NC group, signifying a reduction in connectivity strength across different nodes within the CJE brain entropy network among patients.

Overall, this paper proposes a cross-association entropy method based on *Z* score normalization for calculating the connections between brain regions. The advantage of this method is validated through comparison with traditional approaches, leading to the following conclusions. (1) The subject-normalized cross-association entropy can serve as a candidate method for constructing brain connectivity analyses. Compared to conventional functional connectivity, this method provides complementary insights into node connectivity and improves classification performance by 4%, 6%, and 7% in SCZ, MCI, and ASD, respectively. (2) The algorithm’s analysis of connection weights and symmetrical connections demonstrates that the distinctive connectivity patterns observed in the caudate nucleus in SCZ, as well as cerebellar connections in ASD and MCI, offer crucial insights into the alterations in brain connectivity associated with these disorders.

### Limitations of the study

This study has limitations. Firstly, the sample size for each disease group was relatively small, which is a significant constraint. Future research endeavors will aim to procure larger sample sizes from each database to enhance the robustness of the findings. Secondly, while the results suggest the efficacy of using cross-joint entropy as a primary method for constructing brain entropy connectivity, our investigation into brain entropy connectivity remains incomplete. This study primarily focused on enhancing classification performance and did not comprehensively validate nonlinear and complex alterations in brain entropy connectivity. Thus, we aspire to delve deeper into these aspects in future research endeavors.

## Resource availability

### Lead contact

Requests for further information and resources should be directed to and will be fulfilled by the lead contact, Haifeng Wu (whf5469@gmail.com).

### Materials availability

This study did not generate new subjects.

### Data and code availability

• Data: We validated the Sub_N+CJE algorithm using three public fMRI datasets: MCI data, ASD data, and SCZ data. The original image data addresses of the three databases are:
  1. The Alzheimer’s Disease Neuroimaging Initiative (ADNI) database: https://adni.loni.usc.edu/,
  2. The autistic brain imaging data exchange (ABIDE) database: http://fcon_1000.projects.nitrc.org/indi/abide/abide_II.html,
  3. The Center for Biomedical Research Excellence (COBRE) Schizophrenia Imaging Database: http://fcon_1000.projects.nitrc.org/indi/retro/cobre.html.
• Code: The code used in this study is publicly available at https://github.com/monk5469/Entropy. The copyright for the code belongs to the School of Electrical & Information Technology, Yunnan Minzu University.
• Additional information: Any additional information required to reanalyze the data reported in this paper is available from the lead contact upon request.

## Acknowledgments

This work is supported by the 10.13039/501100001809National Natural Science Foundation of China (62161052), and the Scientific Research Fund project of in Education Department of Yunnan Province (2024Y432).

## Author contributions

H.W., writing – review and editing, supervision, resources, project administration, methodology, funding acquisition, and conceptualization; S.L., writing – review and editing, writing – original draft, visualization, validation, software, resources, methodology, investigation, formal analysis, data curation, and conceptualization; Y.Z., investigation and formal analysis.

## Declaration of interests

The authors declare no competing interests.

## STAR★Methods

### Key resources table

**Table:** 

REAGENT or RESOURCE	SOURCE	IDENTIFIER
**Deposited data**		

rs-fMRI data	This paper	N/A
Preprocessed rs-fMRI data	This paper	https://github.com/monk5469/Entropy

**Software and algorithms**		

MATLAB2021b	MathWorks	RRID: SCR 001622
SPM12	University College	RRID: SCR 007037
DPABI	V6.1_220101	RRID: SCR_002549
Original code	This paper	https://github.com/monk5469/Entropy

### Experimental model and study participant details

The experimental data in this study comprises rs-fMRI data pertaining to three conditions: mild cognitive impairment, autism, and schizophrenia, all sourced from public databases. Specifically, the datasets originate from the Alzheimer's Disease Neuroimaging Initiative (ADNI) database, available at https://adni.loni.usc.edu/; ASD data from autistic brain imaging data exchange (ABIDE) database at Georgetown university, http://fcon_1000.projects.nitrc.org/indi/abide/abide_II.html; and the Center for Biomedical Research Excellence (COBRE) Schizophrenia Imaging Database, at http://fcon_1000.projects.nitrc.org/indi/retro/cobre.html. Table 5 outlines the pertinent parameters of the aforementioned datasets. Additionally, Table 6 provides the basic demographic statistics of subjects across the three disease groups. In this study, the SCZ dataset comprises 145 subjects, the MCI dataset includes 64 subjects, and the ASD dataset consists of 98 subjects. A 5-fold cross-validation strategy was implemented for each dataset to allocate the subjects into experimental groups. No significant differences were observed in age and sex distribution between the MCI and SCZ groups, while no significant age differences were found between the ASD groups. However, gender differences were present between the ASD groups. As a result, gender was included as a covariate in the classification of ASD to minimize its influence on the outcomes. This study did not recruit any new patients and utilized publicly available datasets. It has been approved by the Yunnan Minzu University Artificial Intelligence and Engineering Ethics Committee(study approval number 20240001).

**Table 5:** rs-fMRI data parameters

	MCI	ASD	SCZ
Name database	ADNI-2	ABIDE	COBRE
Magnetic field strength	3.0 Tesla	3.0 Tesla	3.0 Tesla
Collection equipment	Philips	SIEMENS	SIEMENS
Flip angle	80°	90°	75°
TR	3000 ms	2000 ms	2000 ms
TE	30 ms	15 ms	29 ms
Pixel size	3.3 mm × 3.3 mm	3.3 mm × 3.3 mm	3.75 mm × 3.75 mm
Number of slices	48	33	33
Number of TR	140	180	160

**Table 6:** Basic information statistics of subjects

	ASD/NC		MCI/NC		SCZ/NC	
Male/Female	40/8	29/21	13/19	13/19	58/13	51/23
Chi-square test	$\chi^{2}=7.54$; *p* = 0.006		$\chi^{2}=0$; *p* = 1		$\chi^{2}=2.64$; *p* = 0.1	
Age	13.91–8.12 10.90 ± 2.35	13.79–8.06 10.45 ± 2.88	64∼83 71.88 ± 30.02	66∼86 74.31 ± 22.87	18∼65 38.16 ± 193.04	18∼65 35.82 ± 134.09
T-test	t = 1.4224; *p* = 0.1579		t = −1.8784; *p* = 0.0650		t = −1.1078; *p* = 0.2698	

### Method details

#### Related works of entropy

As the field of brain area connectivity research continues to expand, researchers have proposed various methods for assessing brain area connections beyond the Pearson correlation coefficient. Among these, brain entropy connectivity has emerged as a method garnering widespread attention. Entropy, originally rooted in thermodynamics, quantifies the degree of disorder within a system, known as thermodynamic entropy.^74^ Subsequently, the introduction of information entropy^32^ has offered a novel framework for characterizing the information content and uncertainty of information sources. Given the nonlinear characteristics inherent in brain signals, entropy serves as a quantitative tool capable of effectively capturing the nonlinear dynamics of brain activity, thereby assuming a pivotal role in brain connectivity research.^19^ Typically, the connectivity patterns among brain areas in individuals with brain disorders transition from orderly to disordered states.^75^^,^^76^ In accordance with the nature of entropy, higher levels of disorder within the system correspond to greater entropy values; conversely, as the system becomes more ordered, entropy values decrease. Consequently, brain entropy connectivity values in individuals with brain disorders tend to exhibit higher levels.^77^^,^^78^ The characteristic renders brain entropy connections potentially valuable for the detection and classification of brain diseases.

The concept of information entropy was initially proposed by Shannon and has since undergone further refinement over subsequent decades. In addition to information entropy, a plethora of entropy calculation methods have been introduced, including approximate entropy,^33^ sample entropy,^35^ fuzzy entropy,^37^ and spectral entropy.^40^ Table 4 provides an overview of commonly utilized entropy measures. Various entropy techniques have also found application in the study of brain disorders. For instance, approximate entropy has been employed to discern individual differences in cognitive performance among elderly populations,^18^ while sample entropy has been utilized to investigate the chaotic and stochastic characteristics of fMRI signals in schizophrenia patients' brains,^19^ and fuzzy entropy has been employed to statistically analyze the nonlinear properties and cognitive functions in the brains of Alzheimer’s disease (AD) patients.^20^ Although the studies have yielded promising outcomes in specific brain disorder domains, they primarily focus on signal entropy within individual brain regions rather than the entropy between two signals. This paper denotes this category of entropy as 'single entropy' to differentiate it from the cross-entropy, which will be discussed subsequently.

Compared to single entropy measures, cross-entropy quantifies the mutual disorderliness between two signals, rendering it more suitable for analyzing connectivity among brain regions. Table 4 enumerates cross-entropy variants such as cross-approximate entropy,^46^ cross-sample entropy,^48^ cross-fuzzy entropy,^50^ and mutual information entropy.^43^ Recent studies have integrated physiological signals with cross-entropy methodologies to probe brain area connections. For instance, cross-sample entropy has been employed to construct fMRI-based brain area connections for predicting depression severity,^23^ while cross-approximate entropy has been utilized at the voxel level to assess brain activity complexity and synchrony in normal aging and cognitive decline associated with neurodegenerative diseases.^21^ Additionally, cross-sample entropy has been applied to calculate EEG signal functional connections and analyze brain synchronization across various emotional states.^24^ Furthermore, multi-channel cross-fuzzy entropy has been utilized to construct time-varying networks for identifying changes in brain network connections during general anesthesia.^25^ Moreover, a study employing cross-conditional entropy analyzed the pharmacological effects of drugs on brain connections, demonstrating a decrease in linear characteristics and an increase in non-linear characteristics with varying drug dosages.^22^ However, the studies often concentrate solely on applying specific entropy types to particular diseases or contexts, lacking comprehensive evaluations of different cross-entropy methodologies' impact on constructing brain connectivities and failing to provide clear recommendations for researchers on selecting appropriate entropy methods for brain connectivity construction. Moreover, the potential influence of different signal normalization methods on cross-entropy calculations and subsequent implications for brain disease classification remains unaddressed. Consequently, this paper aims to furnish more comprehensive guidance for future brain connectivity research by assessing the performance of various cross-entropy and normalization techniques in brain disease classification.

#### Problem description

**Figure 10:**
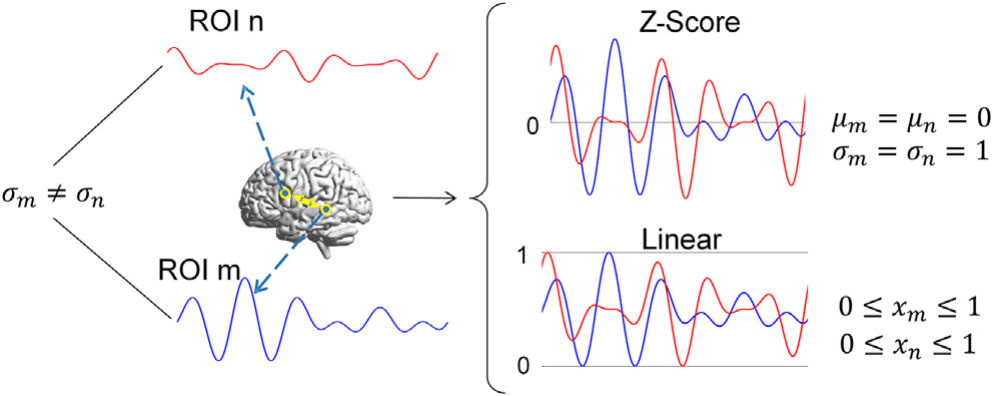
Linear and *Z* Score normalization methods

In the computation of brain connectivity, signal normalization of brain region signals is an indispensable step. Due to variances in physiological attributes and acquisition conditions among subjects, individual differences arise in the amplitudes of obtained brain signals. Moreover, inconsistencies in signal amplitudes may even manifest between different brain regions within the same subject. To mitigate the impact of the amplitude differences on the calculation outcomes of brain region connectivity, normalization becomes a requisite preprocessing step. As depicted in Figure 10, Z-Score normalization and linear normalization stand out as two widely embraced signal normalization techniques. Z-Score normalization entails adjusting the mean of the original signal to 0 and standardizing the variance to 1, whereas linear normalization confines the signal's amplitude range between 0 and 1. In signal correlation analyses, signals with larger amplitudes often yield higher correlation values; thus, Z-Score normalization aids in diminishing the influence of amplitude disparities on correlation calculation outcomes. In entropy computations, establishing the signal's probability distribution is imperative. Linear normalization standardizes the signal's interval range, thereby streamlining the process of probability distribution acquisition. However, while these two normalization methods mitigate the influence of amplitude differences within a single brain region on brain connectivity calculations, they also inadvertently remove amplitude differences between two or more brain regions. Nonetheless, the discrepancy may not be significant when analyzing brain disease groups and controls. The connectivity characteristics among groups of brain regions hold paramount importance; for instance, alterations in signal amplitude within a specific brain region may serve as a pivotal indicator in discerning its involvement in a particular cognitive task. Hence, this paper's primary focus is to propose a normalization method capable of standardizing the signal scale while preserving amplitude differences between signals, thereby more accurately delineating the characteristics of brain region connectivity.

**Figure 11:**
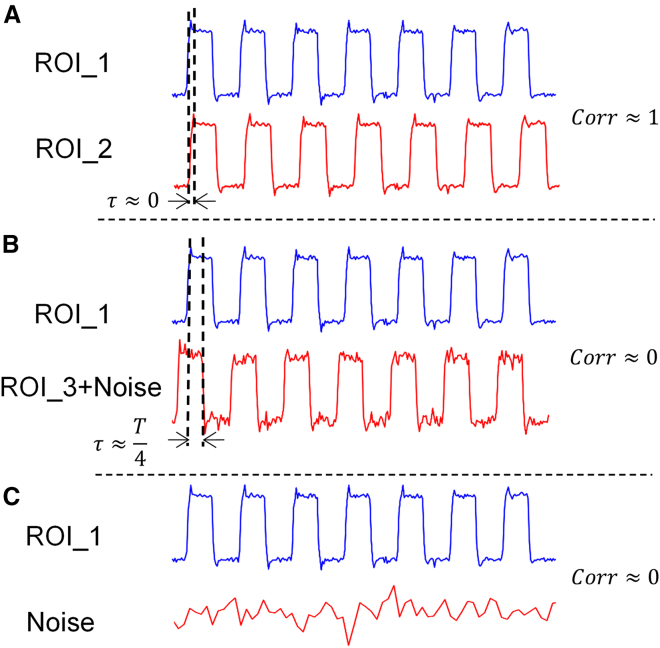
Pearson correlation is examined under three conditions (A) Between two synchronous ROI signals, yielding a correlation value of 1. (B) Between two ROI signals with a quarter-cycle time difference (where in one ROI signal contains noise), resulting in a correlation value of 0. (C) Between an ROI signal and noise, also yielding a correlation value of 0.

Furthermore, this paper addresses the quantification methodology of brain region connectivity, a pivotal aspect of investigation. Traditional approaches to quantifying brain region connectivity, such as the Pearson correlation coefficient, primarily assess the synchronicity between brain regions. As illustrated in Figure 11, during cognitive tasks, BOLD signals within brain regions may exhibit diverse response patterns. In Figure (A), both brain regions exhibit synchronized responses to the task, yielding a Pearson correlation value close to 1; conversely, in Figure (B), while both brain areas respond to the task, their activities are asynchronous, potentially resulting in a correlation value close to 0. Pearson correlation effectively distinguishes synchronous brain area activities in Figure (A) from asynchronous activities in Figure (B). However, in Figure (C), one brain region exhibits a significant response to the task while the other does not. In such cases, the Pearson correlation between the two may also approach 0. This suggests that Pearson correlation might not adequately differentiate between low correlation values due to asynchrony in (B) or independence in (C). Consequently, this paper delves into whether the independence and non-synchrony of brain regions can be discerned by introducing brain entropy connectivity, thereby compensating for Pearson correlation's limitations in depicting brain region connectivity information. Notably, various calculation methods exist for both single entropy and cross-entropy, and determining which method is suitable for specific signals or diseases necessitates focused inquiry.

#### Algorithm

##### Subject normalization algorithm

**Figure 12:**
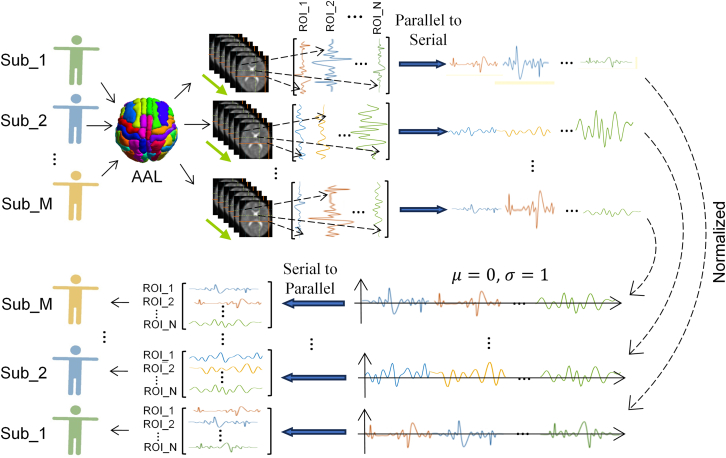
Flow chart of subject normalization algorithm

In this section, we initially introduce the proposed subject normalization algorithm, depicted in Figure 12. Firstly, the BOLD signals from multiple Regions of Interest (ROIs) are extracted from the subject's fMRI data, with ROI selection facilitated by the Automatic Anatomical Labeling Atlas (AAL) template.^79^ Secondly, the ROI signals are concatenated end-to-end and merged into a one-dimensional sequential signal (parallel to serial), enabling comprehensive processing of multiple brain region signals as a unified entity. Subsequently, Z-Score normalization is applied to the amalgamated sequential signal, ensuring a mean of 0 and unit variance, thereby rectifying discrepancies in signal amplitudes across different subjects. Finally, the normalized signals are reinstated to each ROI in their original concatenation sequence, yielding the ultimate normalized signal for each ROI (serial to parallel). The proposed normalization method excels in rectifying signal amplitude disparities between subjects while preserving amplitude variations among different ROI signals within each subject. Subsequently, we provide a detailed elucidation of the specific steps comprising the normalization method.

Let $x_{n}$ be the BOLD signal vector extracted from the n-th ROI of a subject, n=1,2,…N, expressed as

(Equation 1) $$x_{n}={\left[\alpha_{n}\left(1\right),\alpha_{n}\left(2\right),\ldots \alpha_{n}\left(T\right)\right]}^{T}\in R^{T\times 1}$$

where ${\left[\cdot \right]}^{T}$ represents transposition, $\alpha_{n}\left(t\right)$ represents the signal at the t-th Time of Repetition(TR), t=1,2…T. The N ROI signals of the subject are concatenated into a vector signal, expressed as

(Equation 2) $$X={\left[{x_{1}}^{T},{x_{2}}^{T},\ldots {x_{N}}^{T}\right]}^{T}\in R^{NT\times 1}$$

Then, perform Z-Score normalization on the vector signal X, then

(Equation 3) $$U=\frac{X-\mu_{X}}{\sigma_{X}}$$

where $\mu_{X}$ and $\sigma_{X}$ are the mean and variance of the vector X respectively. Since the signal X before normalization is concatenated from the ROI signals, the signal U after normalization can also be regarded as a concatenated signal, that is

(Equation 4) $$U={\left[{u_{1}}^{T},{u_{2}}^{T},\ldots {u_{N}}^{T}\right]}^{T}\in R^{NT\times 1}$$

Then the normalized signal of each ROI will also be reinstated from the vector U, so the n-th normalized ROI signal is

(Equation 5) $$u_{n}=U\left(\left(n-1\right)T+1:nT\right)\in R^{T\times 1}$$

where U(n:m) represents a new vector composed of the n-th to m-th elements of U.

##### Cross-joint entropy brain connectivity algorithm

**Figure 13:**
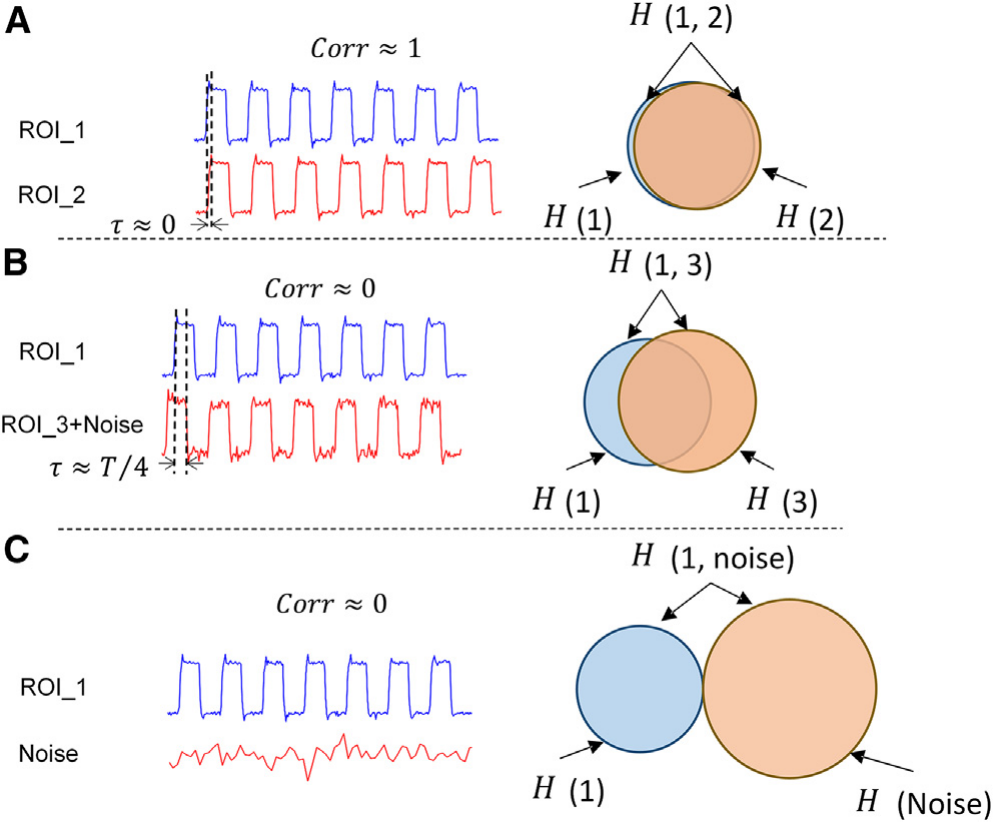
Cross-joint entropy is exampled across three scenarios (A) Between two synchronized ROI signals, yielding the lowest joint entropy. (B) Between two ROI signals with a one-quarter period time difference (one ROI signal containing noise), resulting in the second lowest joint entropy. (C) Between an ROI signal and noise, with the joint entropy being the highest.

This section introduces the utilization of cross-joint entropy for constructing connectivity between brain regions. To elucidate the role of cross-joint entropy in BOLD signal analysis, Figure 13 provides several examples showcasing cross-joint entropy outcomes between different signal types. The examples encompass two synchronous BOLD signals (Figure 13A), two asynchronous orthogonal BOLD signals (with a period of T/4) (Figure 13B), and the outcomes of the BOLD signal combined with white noise (Figure 13C). From the results, it is evident that in cases of synchronization and orthogonality, the cross-joint entropy value between signals is low, whereas in the presence of white noise, the cross-joint entropy value is relatively high. The phenomenon underscores that, compared to the Pearson correlation coefficient, although cross-joint entropy may not exhibit distinct differences between synchronous and asynchronous signals, it demonstrates a unique advantage in distinguishing between signal independence and non-independence. As resting state brain signals can exhibit other forms beyond just synchronous and asynchronous patterns, cross-joint entropy and Pearson correlation consequently exhibit some complementary aspects in constructing brain connectivity. Subsequently, we outline the specific process of calculating brain connectivity using cross-joint entropy.

Let $u_{m}$ and $u_{n}$ be the m-th and n-th ROI signals of a subject after normalization respectively, then the joint information entropy of the two can be expressed as

(Equation 6) $$H\left(u_{m},u_{n}\right)=-\sum_{l=1}^{L}\sum_{l^{\prime }=1}^{L}p\left(\mu_{l}^{\left(m\right)},\mu_{l^{\prime }}^{\left(n\right)}\right)\log\;p\left(\mu_{l}^{\left(m\right)},\mu_{l^{\prime }}^{\left(n\right)}\right)$$

where $p\left(\mu_{l}^{\left(m\right)}\right)$ is the probability when $u_{m}$ take the l-th value. To estimate the joint probability $p\left(\mu_{l}^{\left(m\right)},\mu_{l^{\prime }}^{\left(n\right)}\right)$ and improve its calculation efficiency, the histogram rectangular grid^80^ can be used for calculation. According to the sequence length of $u_{m}$ and $u_{n}$ , divide it into a rectangular grid of I×J, each grid size is Δx×Δy, and the coordinates are (i,j). Express the probability that the values of $u_{m}$ and $u_{n}$ fall in the network (i,j) as

(Equation 7) $$\begin{array}{c} p_{ij}=\iint_{\text{cell}\left(i,j\right)}f\left(u_{m},u_{n}\right)dmdn\approx f\left(x_{i},y_{j}\right)\Delta x\Delta y \end{array}$$

where $\left(x_{i},y_{j}\right)$ represents the center of each unit, and f(·) represents the estimate of the probability density distribution function (PDF). If the PDF is approximately constant within each unit, the approximate value of $H\left(u_{m},u_{n}\right)$ is

$$H\left(u_{m},u_{n}\right)\approx -\sum_{i,j}\Delta x\Delta y\cdot f\left(x_{i},y_{j}\right)\log\;f\left(x_{i},y_{j}\right)$$

(Equation 8) $$\begin{array}{c} \approx -\sum_{i,j}p_{ij}\left(\log\;p_{ij}-\log\left(\Delta x\Delta y\right)\right) \end{array}$$

If the summation boundary of i and j is omitted, and then $k_{ij}/N$ ($k_{ij}$:the number of samples observed in unit (i,j), N: the total number of samples) is approximately substituted for $p_{ij}$, substituted into (8) will have the estimated function of $H\left(u_{m},u_{n}\right)$ expressed as

(Equation 9) $$\begin{array}{c} \hat{H}\left(u_{m},u_{n}\right)=-\sum_{i,j}\frac{k_{ij}}{N}\left(\log\frac{k_{ij}}{N}\right)+\log\left(\Delta x\Delta y\right) \end{array}$$

Given that the joint entropy in Equation 9 is not derived directly from the calculation method outlined in Equation 6, it is no longer referred to as joint information entropy. Following the naming convention established in literature,^27^^,^^53^ this paper adopts the term 'cross-joint entropy' to denote the estimate of joint entropy. Similarly, by substituting the formulas for conditional entropy and approximate entropy from Table 4 into Equation 6, the corresponding cross-conditional entropy and cross-approximate entropy can be derived. However, the derivations will not be elaborated upon further here.

##### Brain entropy connectivity and brain disease classification

After computing the joint entropy between two ROIs, a subject's ROI entropy connectivity matrix is constructed. The constructed matrix is then utilized to classify individuals into brain disease and healthy groups, thus assessing the efficacy of entropy connectivity in classification. The subsequent process is outlined below.

After calculating the cross-joint entropy of N ROIs of a subject, the normalized brain region entropy connectivity matrix can be obtained as

(Equation 10) $$H=\left[\hat{H}\left(u_{m},u_{n}\right)\right]\in R^{N\times N}$$

Given the matrix's symmetric nature and the fact that the diagonal represents the entropy of the signal itself, values above the diagonal are extracted to form a feature vector for classification, expressed as

(Equation 11) $$U=UT\left(H\right)\in R^{\frac{N\left(N-1\right)}{2}\times 1}$$

The function UT(·) represents taking the column vector composed of triangular elements above the diagonal of the matrix. Then, k-fold cross-validation is used to complete the brain disease group classification of brain entropy connectivity, let X be the set composed of all J subjects, and divide the set into $X^{S}$ and $X^{T}$, so that

(Equation 12) $$(X^{S}\cup X^{T}=X)\;\&\;\left(X^{S}\cap X^{T}=0\right)\;\&\;\left(\frac{\left|X^{S}\right|}{\left|X^{T}\right|}=k-1\right)$$

Introducing the symbol j to represent the j-th subject, let $U_{j}$ in (15) and its label $y_{j}$ form a new cell variable

(Equation 13) $$X_{j}=<U_{j},y_{j}>$$

From (6), we form a training set S and a test set T, expressed as

(Equation 14-a) $$S=\left\{X_{j}|j\in X^{S}\right\}$$

(Equation 14-b) $$T=\left\{X_{j}|j\in X^{T}\right\}$$

We first train a classifier $f_{c}\left(\cdot \right)$ in the training set to satisfy

(Equation 15) $$y_{j}=f_{c}\left(U_{j}\right),j\in X^{S}$$

then, $U_{j}$ in the test set is passed through the classifier to obtain the classification label

(Equation 16) $${\hat{y}}_{j}=f_{c}\left(U_{j}\right),j\in X^{T}$$

compare it with the expected label $y_{j}$ to obtain the final classification accuracy. The Algorithm steps are shown in Table 7.

**Table 7:** Algorithm steps

input: N ROI signals of subjects $x_{n}$, n=1,2,…N,number of subjects J output: The test label ${\hat{y}}_{j}$ of the j-th subject
step: ①Normalization: Calculate the subject’s normalized signal $u_{n}$ from (Equation 1), (Equation 2), (Equation 3), (Equation 4), (Equation 5), ②Cross-joint entropy: Calculate the cross-joint entropy between subject ROIs by (Equation 6), (Equation 7), (Equation 8), (Equation 9), ③Entropy connectivity: The entropy connectivity vector U is obtained from (Equation 10), (Equation 11), ④Cross-validation: The training set S and the test set T are composed of 12-14, 15 completes the training, (16) obtains the test result ${\hat{y}}_{j}$

#### Data preprocessing

The data in this paper was preprocessed using the data processing & analysis of brain imaging (DPABI)^81^ toolbox. The download address is http://rfmri.org/DPABI, the version is DPABI_V6.1_220101. In the pre-processing, some data which cannot be correctly registered with structural image and functional image are eliminated. The main steps are as follows:

• Convert the original data to NIFIT format and remove the first 10 frames of unstable images;
• Determine the number of slices and use interlayer scanning to avoid delays in scanning adjacent layers;
• Carry out head movement correction to avoid interference from factors such as head movement, breathing and heartbeat;
• Carry out spatial standardization to eliminate differences in brain structure between subjects;
• Remove noise covariates and linear drift, adjust head movement parameters, and filter out low-frequency offset and high-frequency noise, where the filtering range is 0.01∼0.1Hz;
• Use AAL116 template^79^ to extract the original ROI signal.

Other detailed preprocessing steps for data collection of different equipement can be seen in Table S5.

#### Experimental methods and classification

This experiment considers the following normalization methods, respectively:

• ROI_N: Each ROI signal of a subject is normalized using the Z-Score method^30^ so that its mean is 0 and its variance is 1;
• Sub_N: For the method proposed in this article, see (1-5);
• ROI_NM: The linear normalization method^31^ is used for each ROI signal of each subject, so that the maximum value is 1 and the minimum value is 0.
• Sub_NM: After concatenating all the ROIs of each subject, the linear normalization^31^ method is used. The maximum value of the signal after concatenating and normalization is 1 and the minimum value is 0.
• No_N: The original ROI signals of each subject were used to construct brain connectivity without any normalization method.

When analyzing brain connectivity methods, normalization and cross-entropy are primarily juxtaposed for comparison, alongside related methodologies. Table 8 presents the abbreviations of the cross-entropy and normalization methods employed in this analysis.

**Table 8:** Normalized cross-entropy brain area connection method

Abbreviation	Full name	Abbreviation	Full name
CJE	Cross-joint entropy	CSpE	Cross-spectral entropy
CCE	Cross-conditional entropy	CKE	Cross-Kolmogorov entropy
CApE	Cross-approximate entropy	ROI_N	*Z* Score normalization of ROI
CSaE	Cross-sample entropy	Sub_N	*Z* Score normalization of subject
CFE	Cross-fuzzy entropy	ROI_NM	Linear normalization of ROI
CPE	Cross-permutation entropy	Sub_NM	Linear normalization of subject
CDE	Cross-distribution entropy	No_N	Non-normalized

When employing the aforementioned method to construct brain connectivity for classification, a 5-fold cross-validation approach is utilized. Specifically, the dataset is divided into five subsets, with four subsets used as training sets and one as a test set. Each subset serves as the test set once. To mitigate the influence of chance, the final classification accuracy in the experiment is computed as the average of 100 5-fold cross-validation results. The classifier employed is the support vector machine (SVM), with classification accuracy denoted as 'acc' and defined as follows

(Equation 17) $$acc=\frac{TP+TN}{TP+FP+FN+TN}$$

TP is the number of true positives, TN is the number of true negatives, FP is the number of false positives, and FN is the number of false negatives.

**Figure 14:**
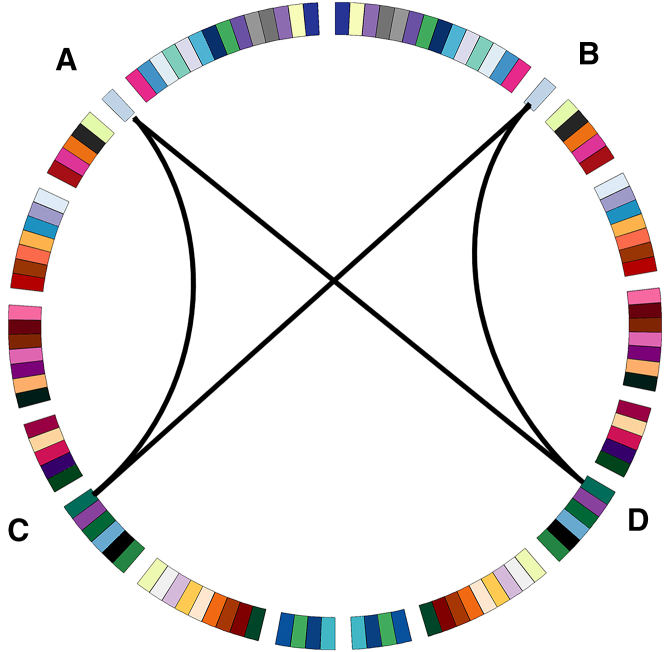
Three symmetrical patterns of brain connectivity "A-D" denotes only the connection points in the graph; for instance, "A-B" represents the connection between point A and point B. The detailed description of the figure is included in the experimental methods and classification section.

To further evaluate the practical significance of the proposed method in the study of brain diseases, this paper examines the symmetry of brain connections across 116 ROIs, with 58 regions in each hemisphere, for the three diseases. The analysis aims to investigate changes in brain connectivity patterns in patients with these conditions. In the experiment, differences between groups in whole-brain connections were identified using two-sample t-tests under various P-values. The brain regions affected by these diseases were localized based on whether the differences in connections were symmetric. Finally, the degree of symmetry variation in 58 pairs of nodes was calculated. The three symmetry phenomena considered in this study are presented in Figure 14, as follows:

• Node connection symmetry: If the left hemisphere ROI A and the right hemisphere ROI B both exchange information with ROI C, and these connections become abnormal when affected by disease, then node connection symmetry is considered to have occurred.
• Intrahemispheric connection symmetry: If ROI A and ROI C in the left hemisphere exchange information, and ROI B and ROI D in the right hemisphere do the same, and both connections become abnormal when affected by disease, then intrahemispheric connection symmetry is considered to have occurred.
• Cross-hemisphere connection symmetry: If the left hemisphere ROI A and the right hemisphere ROI D exchange information, while the right hemisphere ROI B and the left hemisphere ROI C exchange information at the same time, when the disease affects these two connections, then the cross-hemisphere connection symmetry can be considered to have occurred.

ROI proportion of symmetric connections (psc) is defined as

(Equation 18) $$psc=\frac{{TP}^{\prime }}{\text{num}\left(\text{ROI}\right)\times 2-4}$$

where the numerator ${TP}^{\prime }$ represents the number of connections exhibiting symmetry, while the denominator indicates the total number of connections associated with the selected ROI. To eliminate the influence of self-connections and cross-connections involving the selected ROI, these four items are excluded from the total count of connections.

This experiment was conducted under the Windows 11 Professional 64-bit operating system, This experiment was conducted under the Windows 11 Professional 64-bit operating system. The CPU is 11th Gen Intel(R) Core(TM) i7-11800H @ 2.30GHz, the GPU is NVIDIA GeForce RTX 3050 Ti, and the memory is 16GB. All algorithm models are constructed under the Matlab2021b framework.

In addition, graph features derived from brain connectivities were also extracted in the experiment to characterize the fundamental properties of the network and the distribution of node connectivities. In general, these features were selected from an undirected graph (as functional connectivity is inherently undirected), retaining a total of seven features that exhibit significant differences. The pertinent graph features were extracted using the Brain Connectivity Toolbox (https://sites.google.com/site/bctnet/home), with the feature descriptions provided in Table 9.

**Table 9:** Graph features and description

Features	Describe
Global efficiency	The reciprocal of the average shortest path length
Modular	Degree of subdivision into groups
Isogamy	Correlation coefficient between the degrees of all nodes at both ends of the line
Average traffic	The average value of the node’s flow coefficient
Node strength	The sum of the weights of the links connected to the node
Clustering coefficient	Node aggregation degree coefficient
Participation coefficient	A measure of the diversity of inter-module connections at a single node

### Quantification and statistical analysis

#### Group analysis

In this study, subjects with three types of diseases were categorized into patient groups and normal control groups. Following data preprocessing using Dpabi(see subsection data preprocessing), the ROI time series matrix signals for each subject were extracted. Subsequently, the proposed subject normalization method was applied to process the data, after which entropy connectivity was calculated for further analysis. Initially, classification accuracy was assessed to identify the method yielding the best classification performance. Next, computational complexity was evaluated to determine the most efficient feature extraction approach. After a comprehensive evaluation, the optimal method (Sub_N+CJE) was identified.

#### Comparative analysis

We conducted a comparative analysis to evaluate the proposed method against traditional approaches from multiple perspectives. First, Sub_N+CJE was shown to exhibit more differential connections by calculating the connections to the central node (PCC) (see Figure 3). Additionally, connection weights were analyzed to identify differences across various brain regions when compared with traditional methods (see Figures 4, 5, and 6). We also examined the functional connection symmetry (see Figure 14) changes in the brain by comparing the brain entropy connections proposed in this study with traditional methods using a two-sample t-test, and we provided results for three diseases under three different significance thresholds. Finally, differences in test results were calculated based on seven graph theory features (with the significance threshold set at p < 0.05) (see Tables 1, 2, and 3).
