# Supplementary material for: Normalization and cross-entropy connectivity in brain disease classification
Source: iScience. 2025 Mar 17;28(4):112226. doi: 10.1016/j.isci.2025.112226 (PMC11999650; doi:10.1016/j.isci.2025.112226)
Supplement: Document S1. Figures S1–S5 and Table S1–S5 [file mmc1.pdf]

iScience, Volume *28*

Supplemental information

Normalization and cross-entropy connectivity in brain disease classification

Haifeng Wu, Shunliang Li, and Yu Zeng

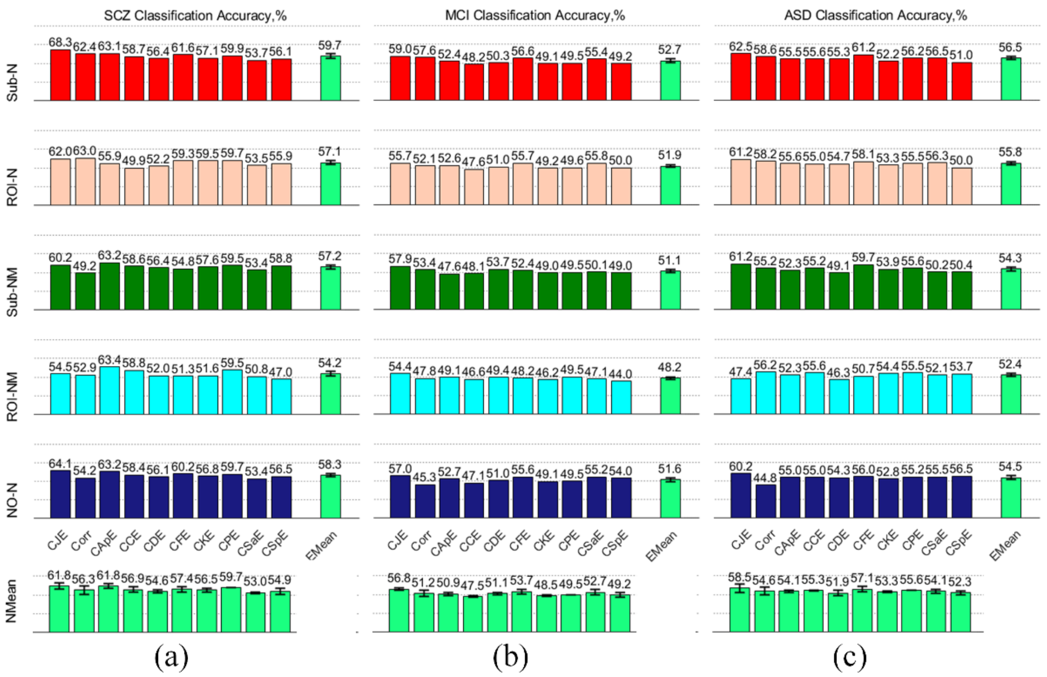

Figure S1. The average classification accuracy of each normalized cross-entropy measure (including correlation) across the three diseases of SCZ, MCI, and ASD is determined by combining 5 normalization methods and 10 cross-entropy measures, in the context of the k-nearest neighbors (KNN) algorithm.

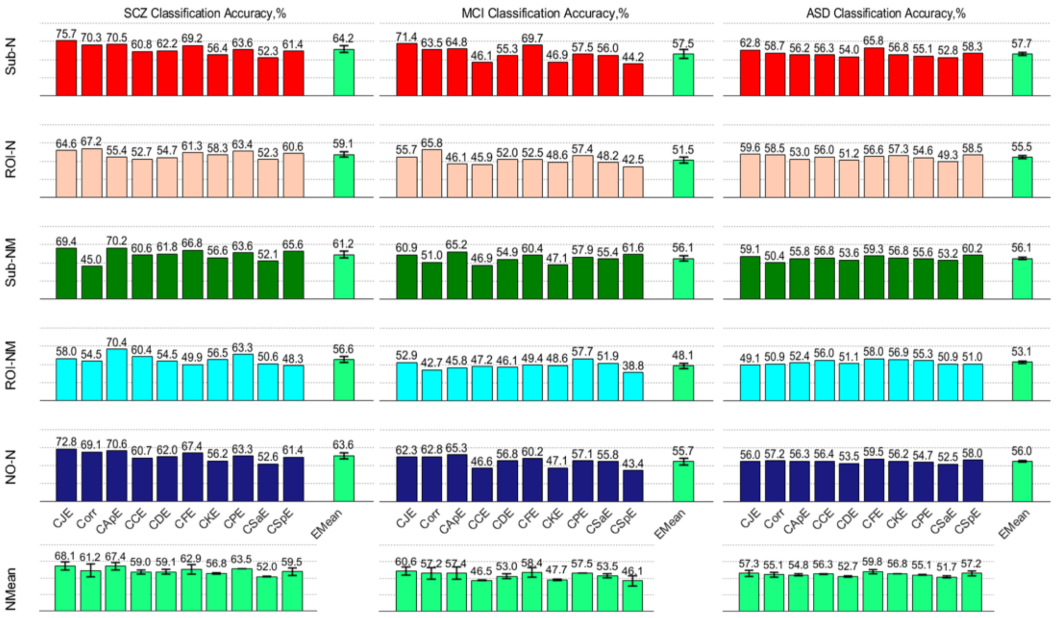

(a) (b) (c)

Figure S2. The average classification accuracy of each normalized cross-entropy measure (including correlation) across the three diseases of SCZ, MCI, and ASD is determined by combining 5 normalization methods and 10 cross-entropy measures, in the context of the random forest algorithm.

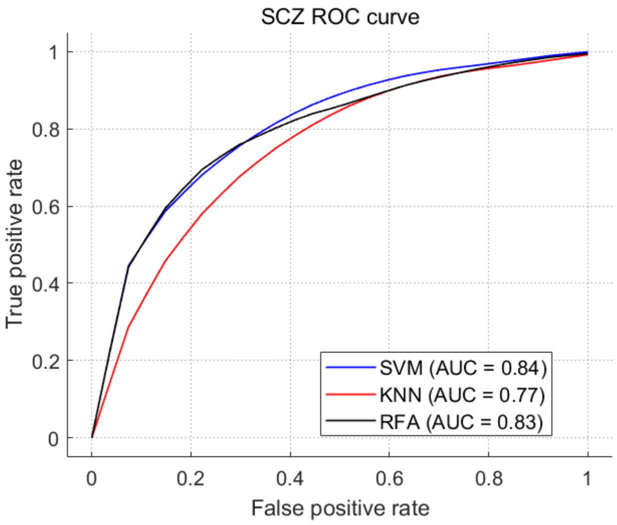

Figure S3. ROC curves and AUC values obtained by three classifiers in SCZ.

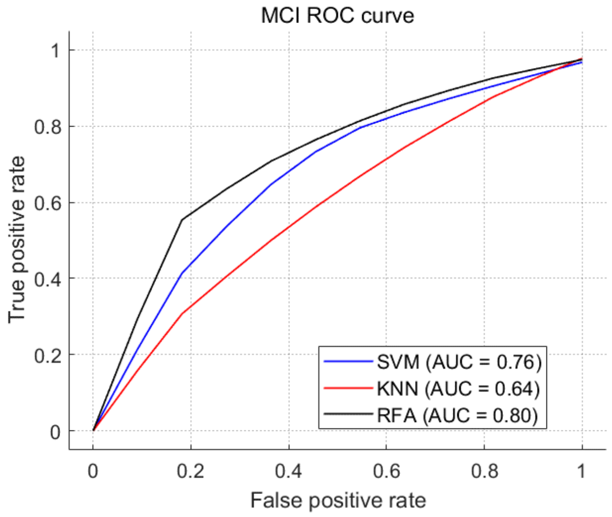

Figure S4. ROC curves and AUC values obtained by three classifiers in MCI.

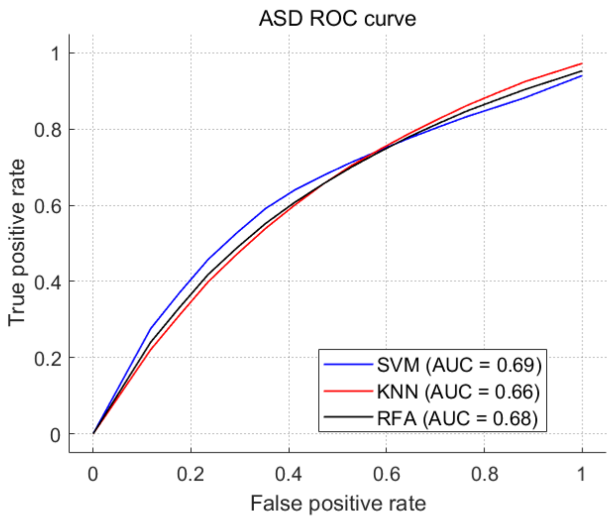

Figure S5. ROC curves and AUC values obtained by three classifiers in ASD.

Table S1. Confusion matrix and precision obtained by three classifiers in SCZ.

SCZ	SVM		KNN		RFA	
	Positives	Negatives	Positives	Negatives	Positives	Negatives
Positives	5260	1740	5750	1250	5618	1382
Negatives	1814	5186	3268	3732	2028	4972
Precision-recall rate	0.74		0.70		0.64	

Note: The value in the confusion matrix is the sum of 100 5-fold cross-validations.

Table S2. Confusion matrix and precision obtained by three classifiers in MCI.

MCI	SVM		KNN		FRA	
	Positives	Negatives	Positives	Negatives	Positives	Negatives
Positives	2115	885	1090	1910	2204	796
Negatives	911	2089	570	2430	956	2044
Precision-recall rate	0.70		0.66		0.65	

Table S3. Confusion matrix and precision obtained by three classifiers in ASD.

ASD	SVM		KNN		RFA	
	Positives	Negatives	Positives	Negatives	Positives	Negatives
Positives	3382	1618	3501	1499	3234	1766
Negatives	1454	2546	1872	2128	1535	2465
Precision-recall rate	0.69		0.65		0.68	

Table S4. The indices and names of the ROIs in the AAL template. The odd and even indices refer, respectively, to the left- and right-hemispheric regions.

Index	ROI label	Index	ROI label
1,2	eReCentral cyrus (eReCG)	3,4	Superior frontal gyrus (dorsal) (SFGdor)
5,6	ORBitofrontal cortex (superior) (ORBsupb)	7,8	Middle frontal gyrus (MFG)
9,10	ORBitofrontal cortext (middle) (ORBmid)	11,12	Inferior frontal gyrus (opercular) (IFGoperc)
13,14	Inferior frontal gyrus (triangular) (IFGtrang)	15,16	ORBitofrontal cortex (inferior) (ORBinf)
17,18	ROLandic operculum (ROL)	19,20	Supplementary motor area (SMA)
21,22	OLFactory (OLF)	23,24	Superior frontal gyrus (middle) (SFGmed)
25,26	ORBitofrontal cortex (medial) (ORBmed)	27,28	RECtus gyrus (REC)
29,30	INSula (INS)	31,32	Anterior cingulate gyrus (ACG)
33,34	Middel cingulate gyrus (MCG)	35,36	eosterior cingulate gyrus (eCG)
37,38	Hippocampus (HIe)	39,40	earahippocampal gyrus (eHG)
41,42	AMYGdala (AMYG)	43,44	CALcarine cortex (CAL)
45,46	CUNeus (CUN)	47,48	LINGual gyrus (LING)
49,50	Superior occipital gyrus (SOG)	51,52	Middle occipital gyrus (MOG)
53,54	Inferior occipital gyrus (IOG)	55,56	FusiForm gyrus (FFG)
57,58	eostCentral gyrus (eoCG)	59,60	Superior parietal gyrus (SeG)
61,62	Inferior parietal lobule (IeL)	63,64	SupraMarginal gyrus (SMG)
65,66	ANGular gyrus (ANG)	67,68	ereCUNeus (eCUN)
69,70	earaCentral lobule (eCL)	71,72	CAUdate (CAU)
73,74	eUTamen (eUT)	75,76	eALlidum (eAL)
77,78	THAlamus (THA)	79,80	HESHl gyrus (HES)
81,82	Superior temporal gyrus (STG)	83,84	Temporal eOle (superior) (TeOsup)
85,86	Middel temporal gyrus (MTG)	87,88	Temporal eOle (middle) (TeOmid)
89,90	Inferior temporal gyrus (ITG)	91–94	Crus I-II of cerebellar hemisphere (Crus)
95−108	Lobule III-X of cerebellar hemisphere (Lobule)	109–116	Lobule I-X of vermis (vermis)

Frontal = (1–16, 19–28, 69–70); insula = (29:30); temporal = (79–90); parietal = (17–18, 57–68); occipital = (43–56); limbic = (31–40); subcortical = (41–42, 71–78); cerebellum = (91–108); vermis = (109–116)

Table S5. Data preprocessing for three different datasets from different databases and acquisition devices.

Preprocessing	Describe
Spatial standardization	The subjects' image data are converted to standard MNI space to reduce spatial differences arising from different devices and resolutions.
Batch effect correction	Using the ComBat batch effect correction tool, covariates related to device type are adjusted to minimize variations in data distribution caused by various devices.
Smoothing processing	Gaussian smoothing is applied to reduce noise and minimize resolution differences associated with different devices.
